# Supplementary material for: Phenylacetyl-/Trolox- Amides: Synthesis, Sigma-1, HDAC-6, and Antioxidant Activities
Source: Int J Mol Sci. 2023 Oct 18;24(20):15295. doi: 10.3390/ijms242015295 (PMC10607876; doi:10.3390/ijms242015295)
Supplement: Supplementary file 1 [file ijms-24-15295-s001.zip › Supplemental Exptal. NMR -IJMS.pdf]

Supplemental ^{1}H and ^{13}C NMR spectra for Compounds 1-20.

Compound #1

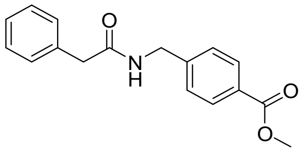

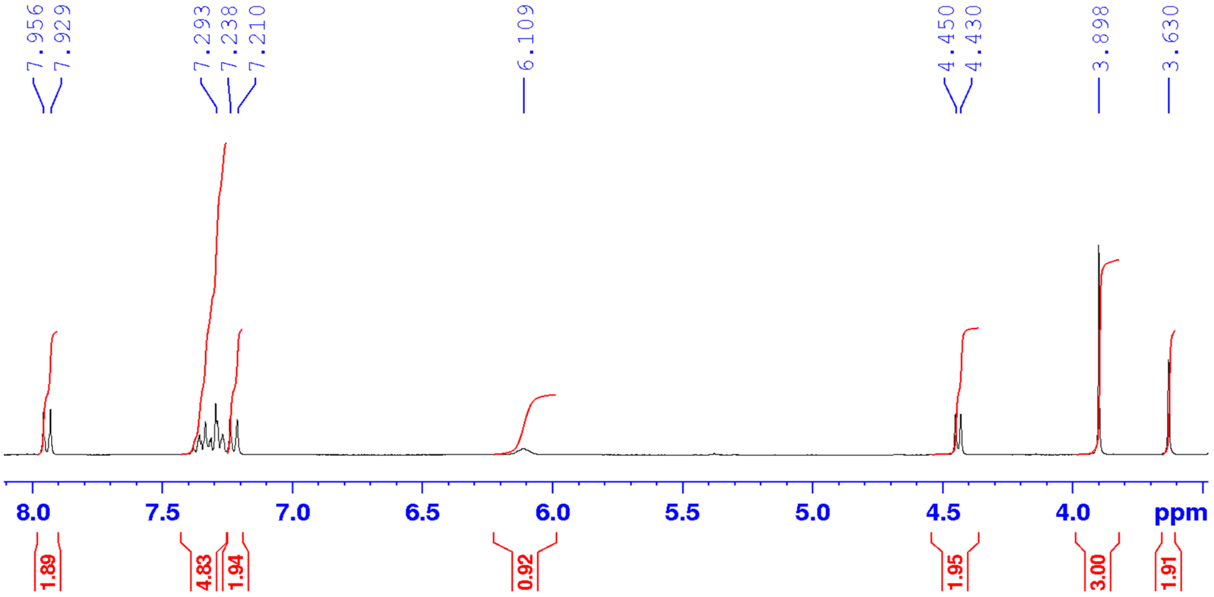

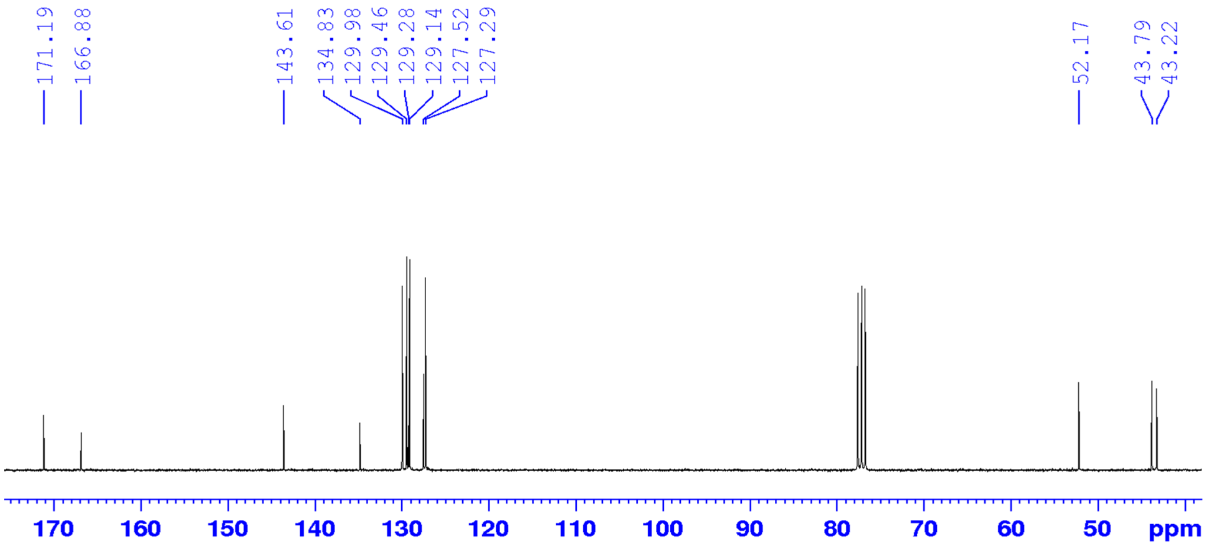

Compound #2

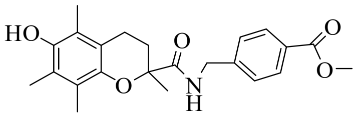

HO
O
N
H
O
O
O

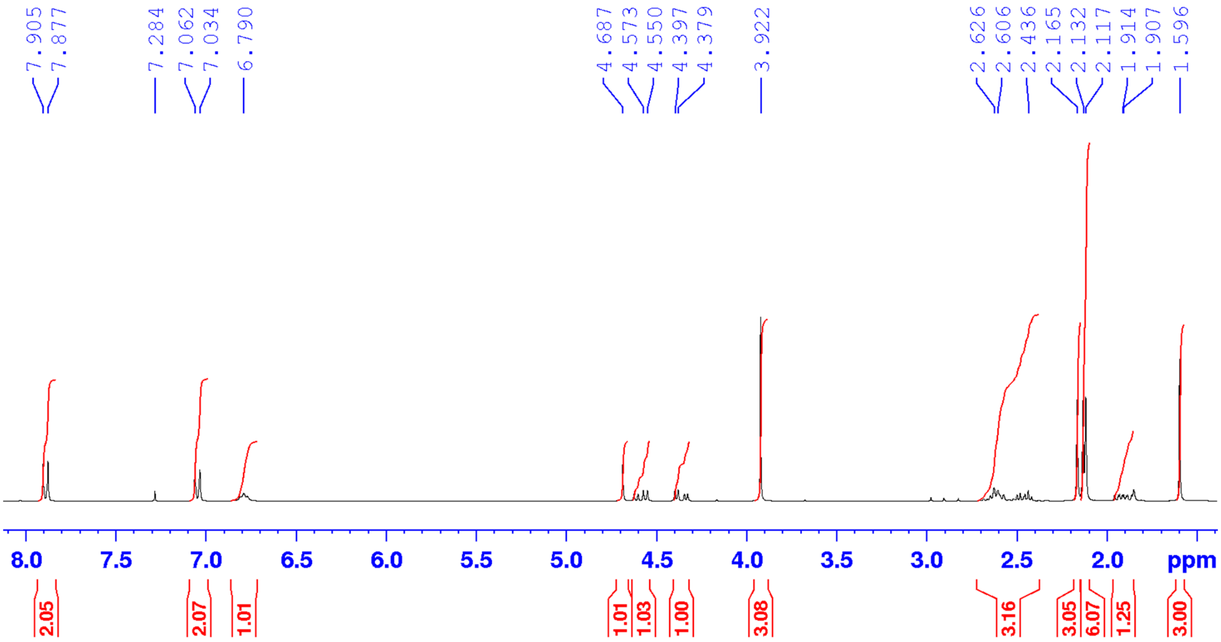

7.905
7.877
7.284
7.062
7.034
6.790
4.687
4.573
4.550
4.397
4.379
3.922
2.626
2.606
2.436
2.165
2.132
2.117
1.914
1.907
1.596
8.0
7.5
7.0
6.5
6.0
5.5
5.0
4.5
4.0
3.5
3.0
2.5
2.0
ppm
2.05
2.07
1.01
1.01
1.03
1.00
3.08
3.16
3.05
6.07
1.25
3.00

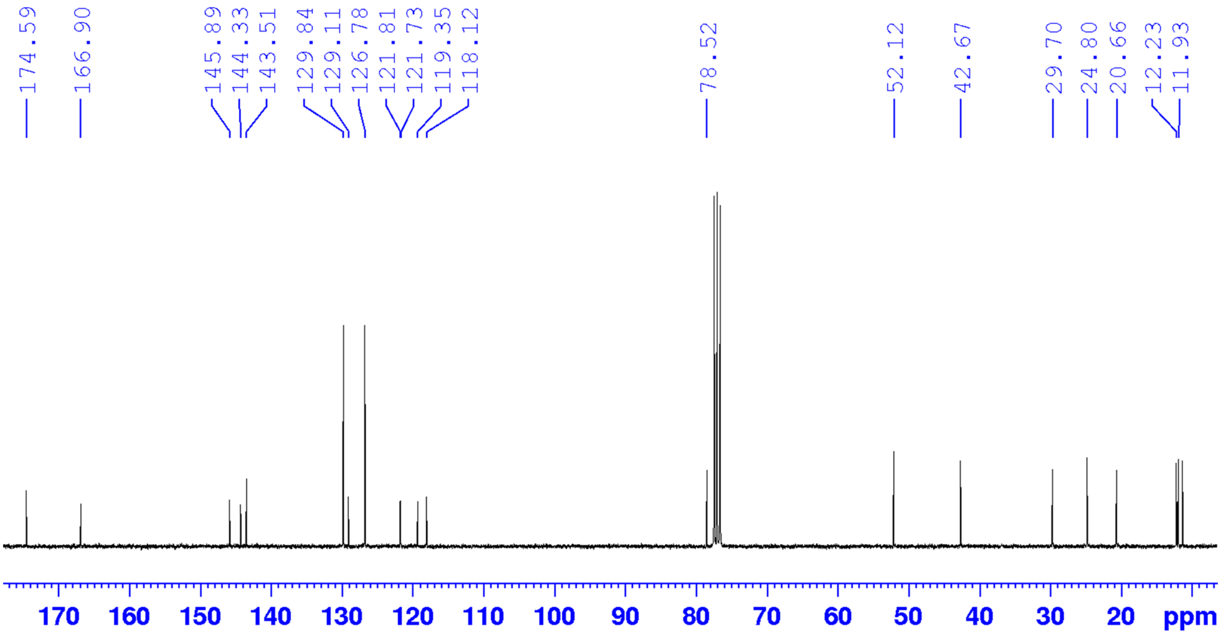

174.59
166.90
145.89
144.33
143.51
129.84
129.11
126.78
121.81
121.73
119.35
118.12
78.52
52.12
42.67
29.70
24.80
20.66
12.23
11.93
170
160
150
140
130
120
110
100
90
80
70
60
50
40
30
20
ppm

Compound #3

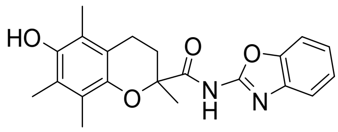
HO
O
O
N
H
O
N

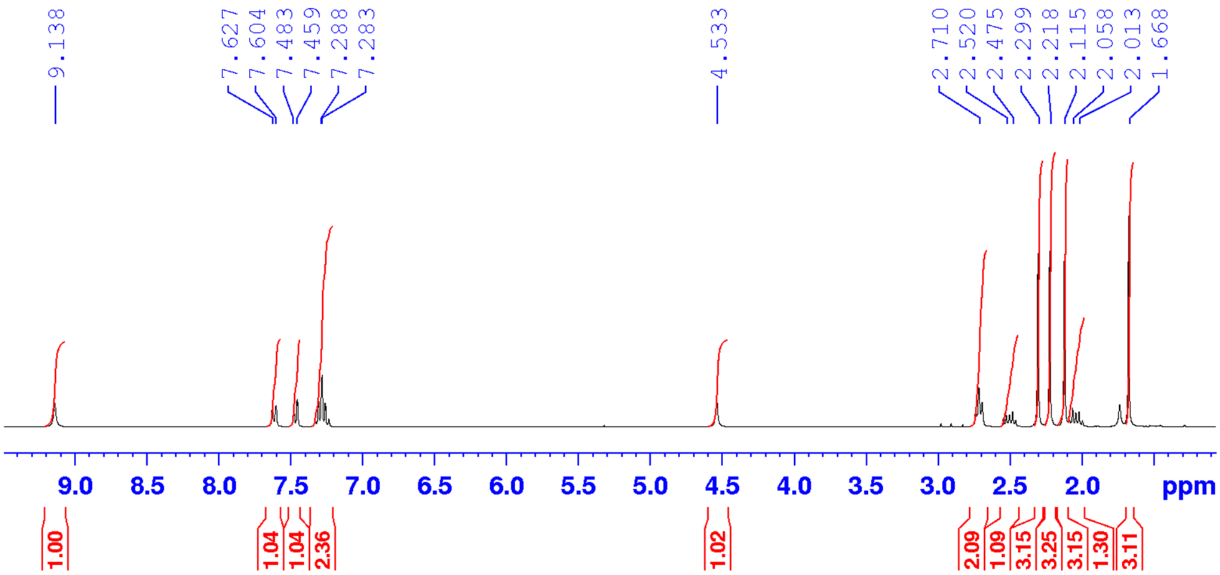
9.138
7.627
7.604
7.483
7.459
7.288
7.283
4.533
2.710
2.520
2.475
2.299
2.218
2.115
2.058
2.013
1.668
9.0 8.5 8.0 7.5 7.0 6.5 6.0 5.5 5.0 4.5 4.0 3.5 3.0 2.5 2.0 ppm
1.00
1.04
1.04
2.36
1.02
2.09
1.09
3.15
3.25
3.15
1.30
3.11

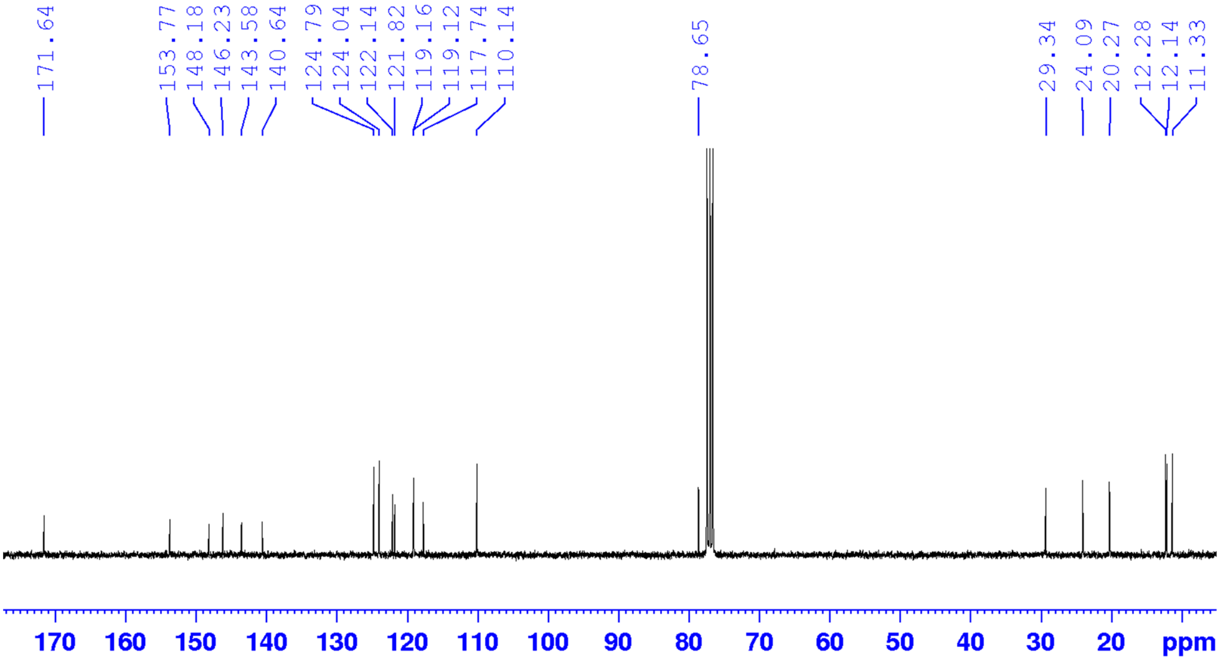
171.64
153.77
148.18
146.23
143.58
140.64
124.79
124.04
122.14
121.82
119.16
119.12
117.74
110.14
78.65
29.34
24.09
20.27
12.28
12.14
11.33
170 160 150 140 130 120 110 100 90 80 70 60 50 40 30 20 ppm

Compound #4

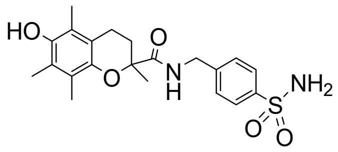
HO
O
N
H
NH2
S
O
O
O

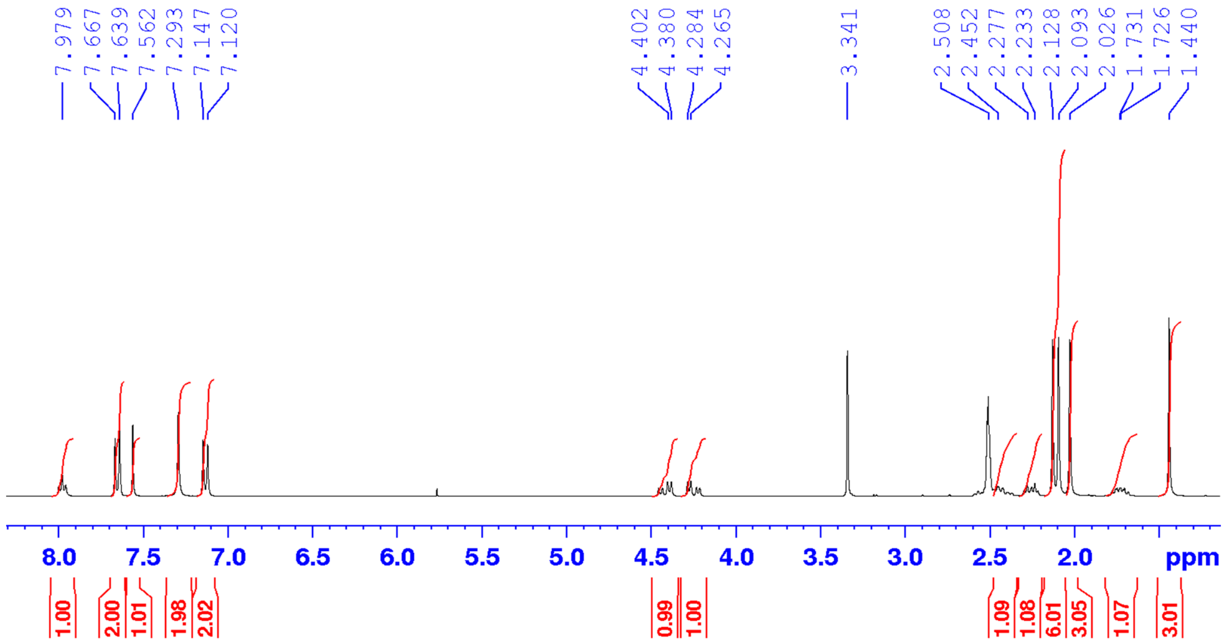
7.979
7.667
7.639
7.562
7.293
7.147
7.120
4.402
4.380
4.284
4.265
3.341
2.508
2.452
2.277
2.233
2.128
2.093
2.026
1.731
1.726
1.440
8.0
7.5
7.0
6.5
6.0
5.5
5.0
4.5
4.0
3.5
3.0
2.5
2.0
ppm
1.00
2.00
1.01
1.98
2.02
0.99
1.00
1.09
1.08
6.01
3.05
1.07
3.01

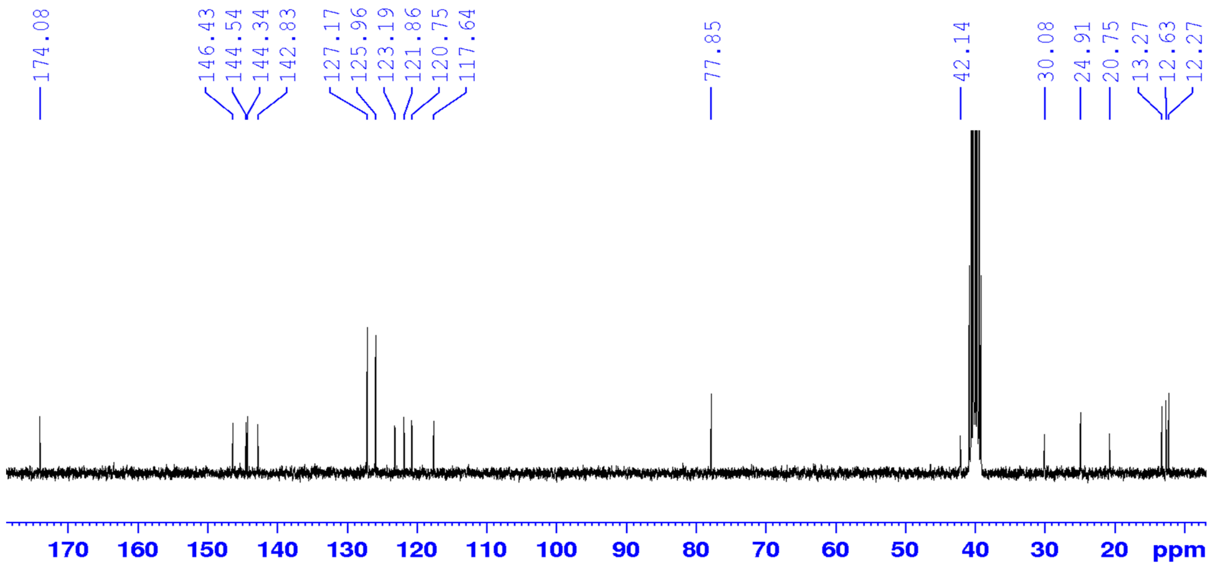
174.08
146.43
144.54
144.34
142.83
127.17
125.96
123.19
121.86
120.75
117.64
77.85
42.14
30.08
24.91
20.75
13.27
12.63
12.27
170
160
150
140
130
120
110
100
90
80
70
60
50
40
30
20
ppm

Compound #5

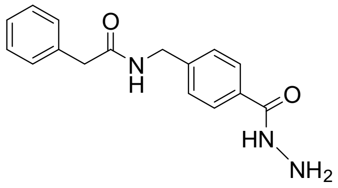
O
N
H
O
HN
NH2

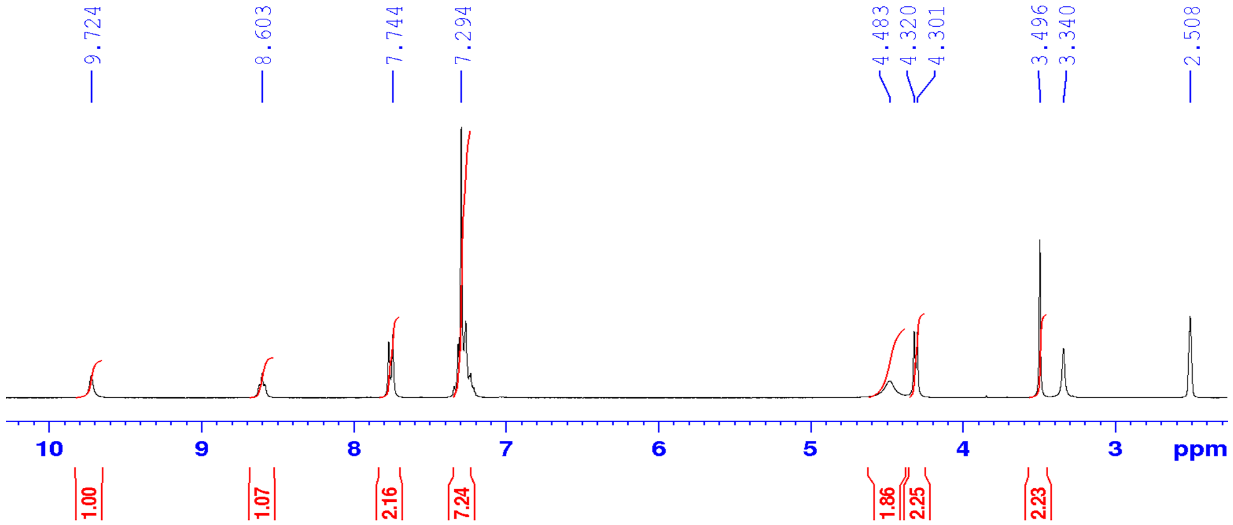
9.724
8.603
7.744
7.294
4.483
4.320
4.301
3.496
3.340
2.508
10
9
8
7
6
5
4
3
ppm
1.00
1.07
2.16
7.24
1.86
2.25
2.23

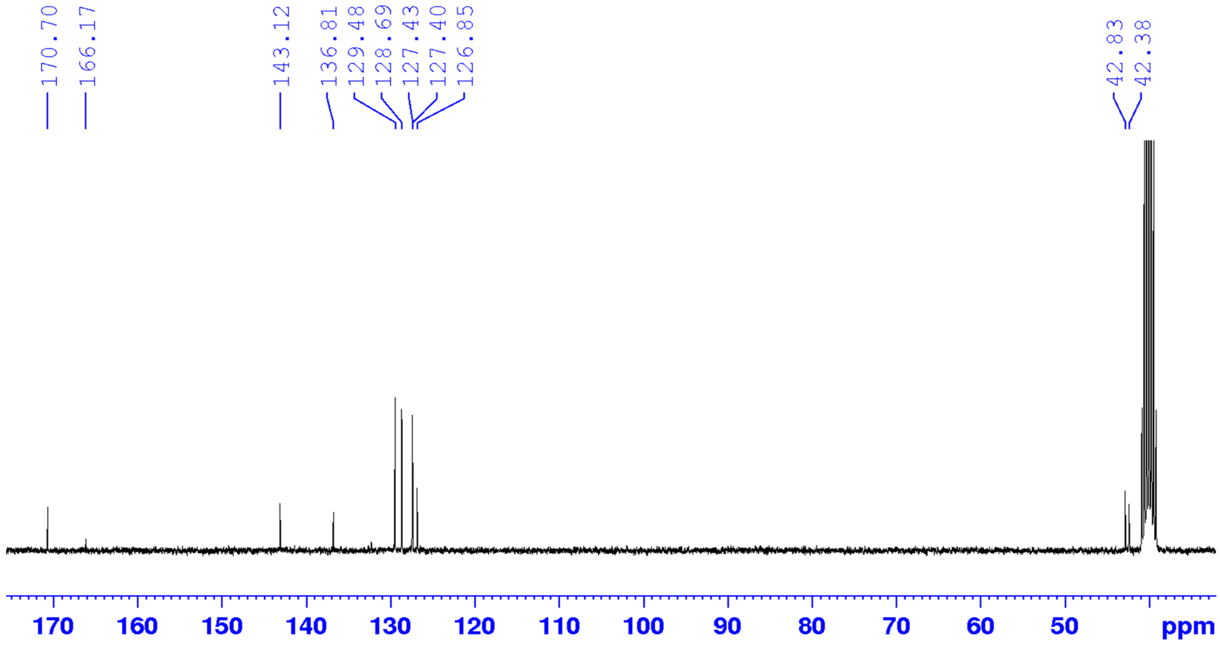
170.70
166.17
143.12
136.81
129.48
128.69
127.43
127.40
126.85
42.83
42.38
170
160
150
140
130
120
110
100
90
80
70
60
50
ppm

Compound #6

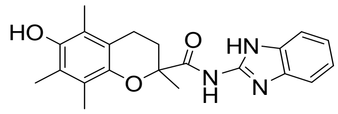
HO
O
HN
N
H
N
O

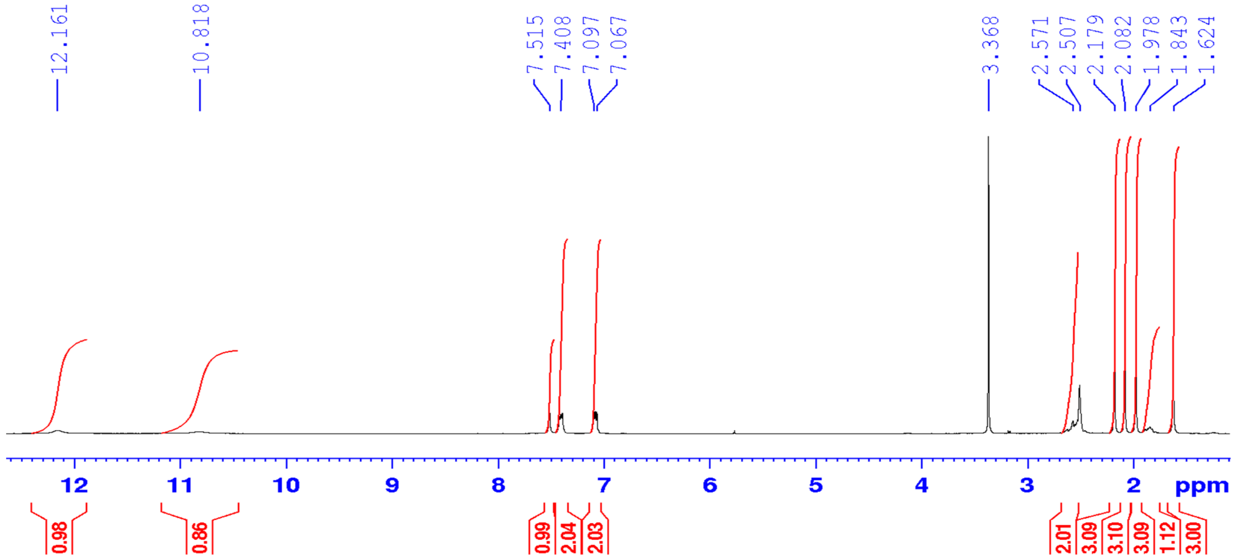
12.161
10.818
7.515
7.408
7.097
7.067
3.368
2.571
2.507
2.179
2.082
1.978
1.843
1.624
12
11
10
9
8
7
6
5
4
3
2
ppm
0.98
0.86
0.99
2.04
2.03
2.01
3.09
3.10
3.09
1.12
3.00

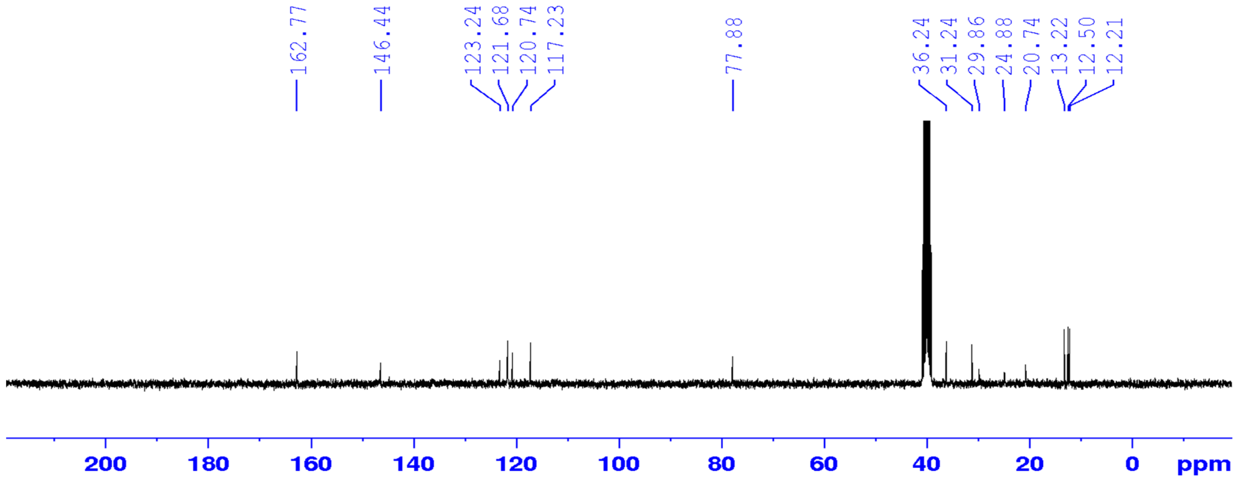
162.77
146.44
123.24
121.68
120.74
117.23
77.88
36.24
31.24
29.86
24.88
20.74
13.22
12.50
12.21
200
180
160
140
120
100
80
60
40
20
0
ppm

Compound #7

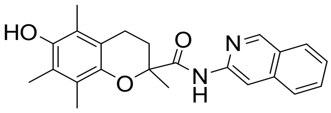
HO
O
O
N
H
N

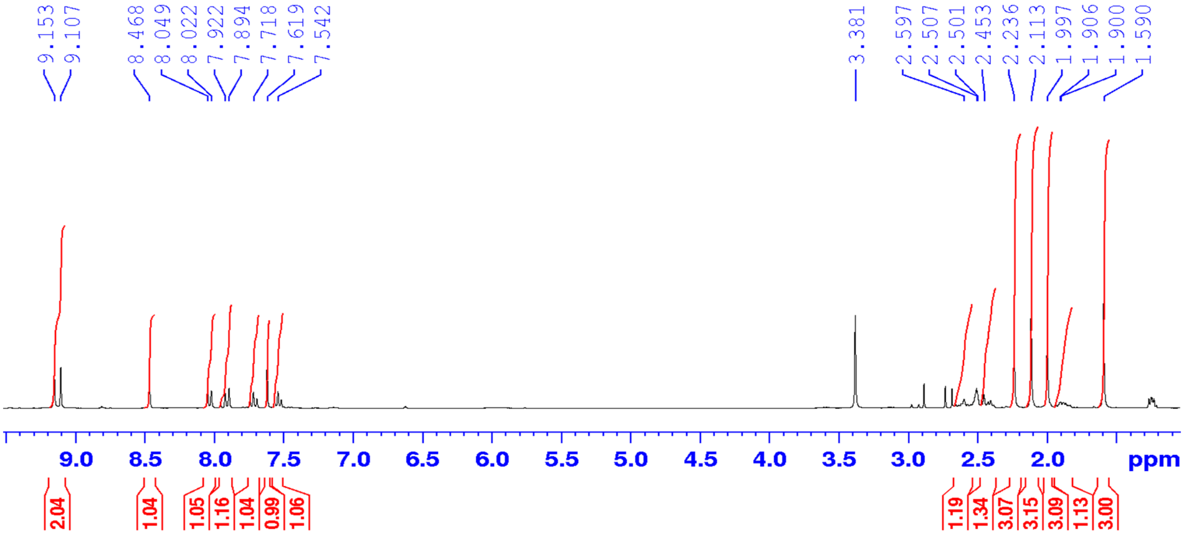
9.153
9.107
8.468
8.049
8.022
7.922
7.894
7.718
7.619
7.542
3.381
2.597
2.507
2.501
2.453
2.236
2.113
1.997
1.906
1.900
1.590
9.0 8.5 8.0 7.5 7.0 6.5 6.0 5.5 5.0 4.5 4.0 3.5 3.0 2.5 2.0 ppm
2.04
1.04
1.05
1.16
1.04
0.99
1.06
1.19
1.34
3.07
3.15
3.09
1.13
3.00

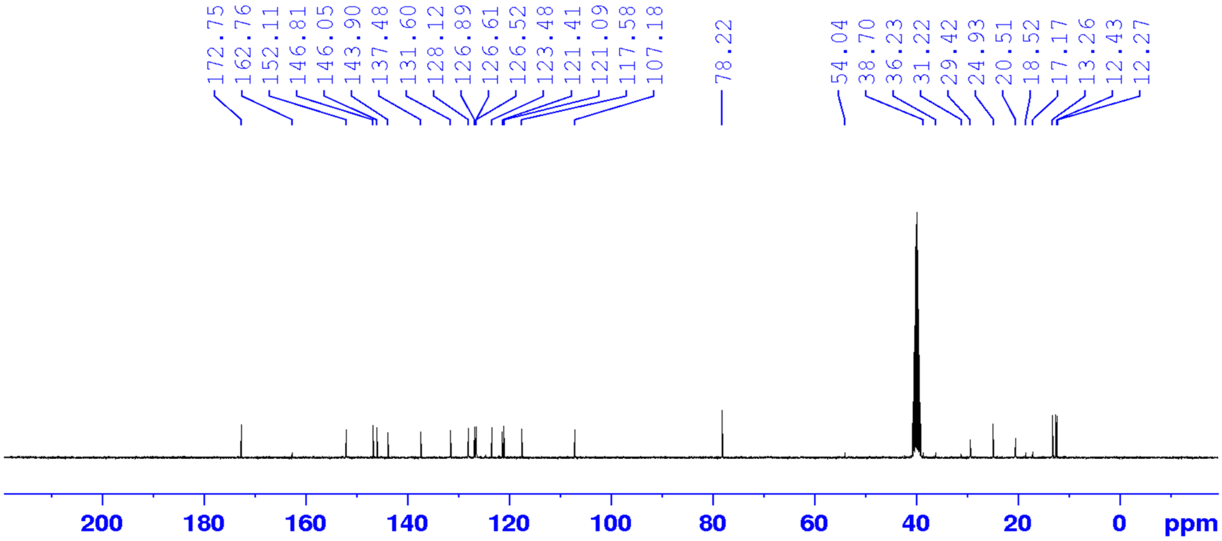
172.75
162.76
152.11
146.81
146.05
143.90
137.48
131.60
128.12
126.89
126.61
126.52
123.48
121.41
121.09
117.58
107.18
78.22
54.04
38.70
36.23
31.22
29.42
24.93
20.51
18.52
17.17
13.26
12.43
12.27
200 180 160 140 120 100 80 60 40 20 0 ppm

Compound #8

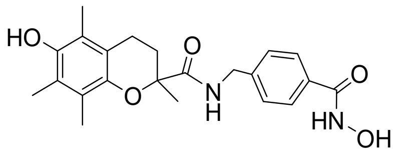
HO
O
N
H
O
HN
OH

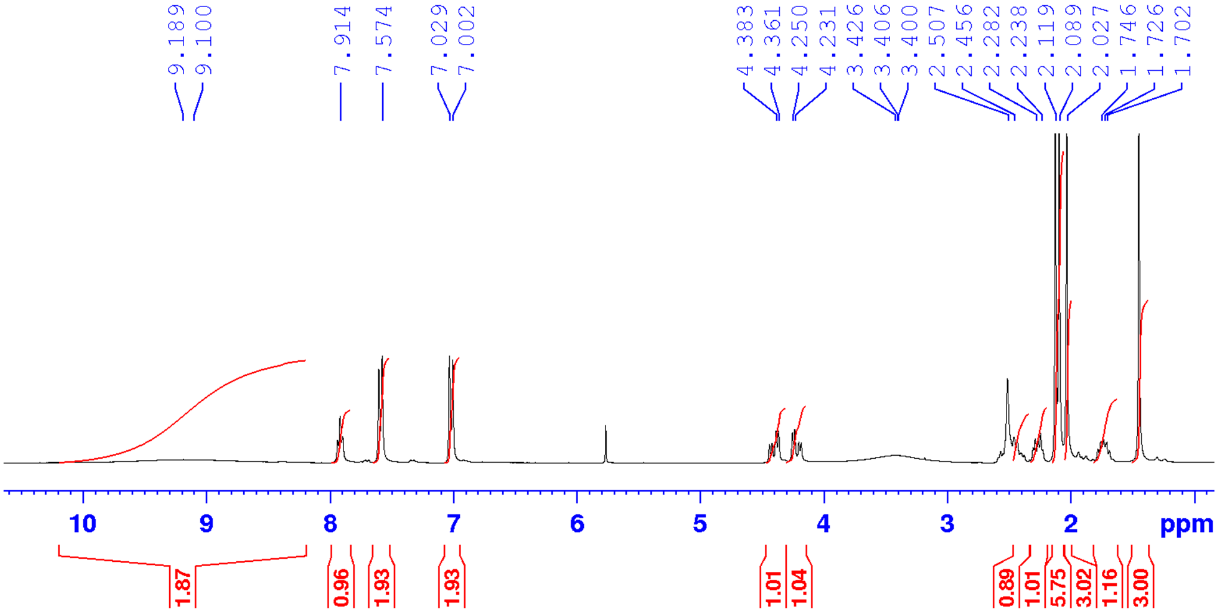
9.189
9.100
7.914
7.574
7.029
7.002
4.383
4.361
4.250
4.231
3.426
3.406
3.400
2.507
2.456
2.282
2.238
2.119
2.089
2.027
1.746
1.726
1.702
10
9
8
7
6
5
4
3
2
ppm
1.87
0.96
1.93
1.93
1.01
1.04
0.89
1.01
5.75
3.02
1.16
3.00

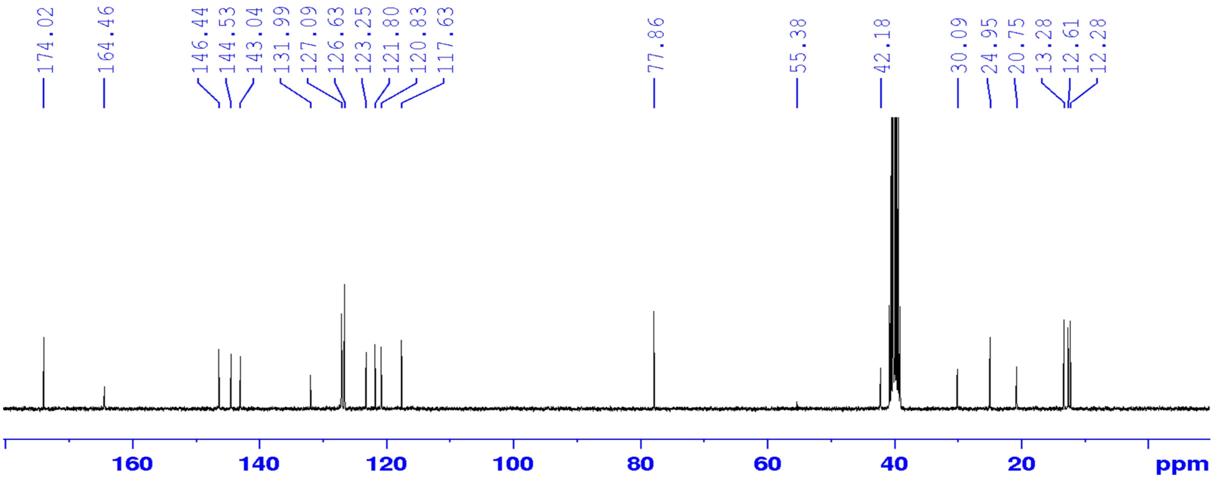
174.02
164.46
146.44
144.53
143.04
131.99
127.09
126.63
123.25
121.80
120.83
117.63
77.86
55.38
42.18
30.09
24.95
20.75
13.28
12.61
12.28
160
140
120
100
80
60
40
20
ppm

Compound #9

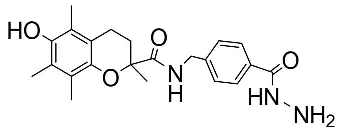
HO
O
N
H
O
O
HN
NH2

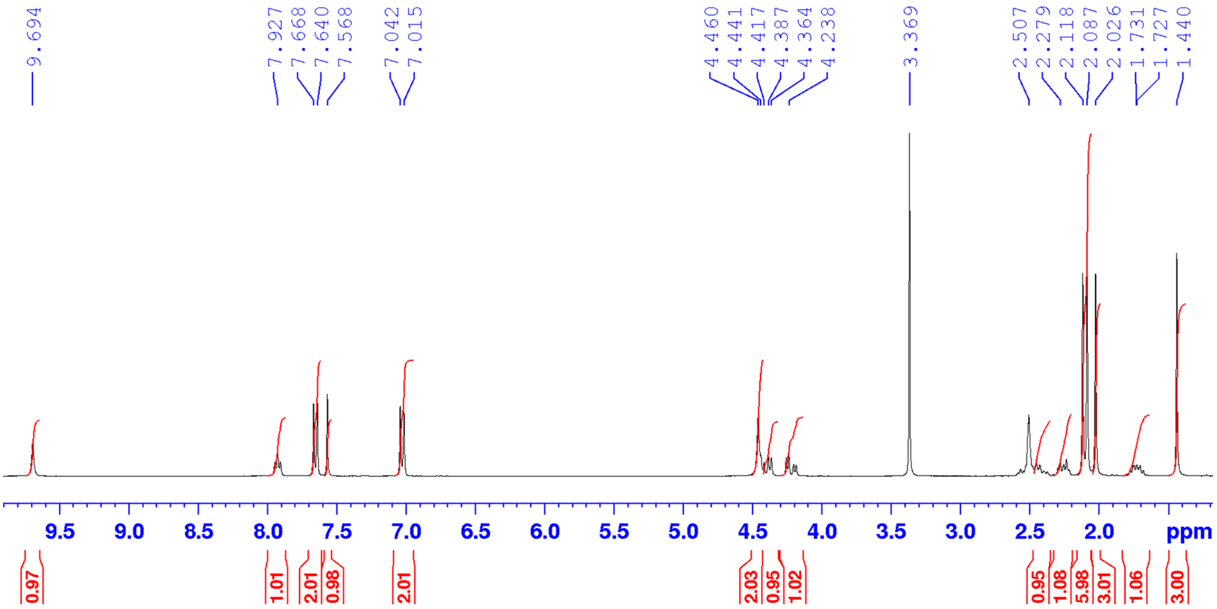
9.694
7.927
7.668
7.640
7.568
7.042
7.015
4.460
4.441
4.417
4.387
4.364
4.238
3.369
2.507
2.279
2.118
2.087
2.026
1.731
1.727
1.440
9.5 9.0 8.5 8.0 7.5 7.0 6.5 6.0 5.5 5.0 4.5 4.0 3.5 3.0 2.5 2.0 ppm
0.97
1.01
2.01
0.98
2.01
2.03
0.95
1.02
0.95
1.08
5.98
3.01
1.06
3.00

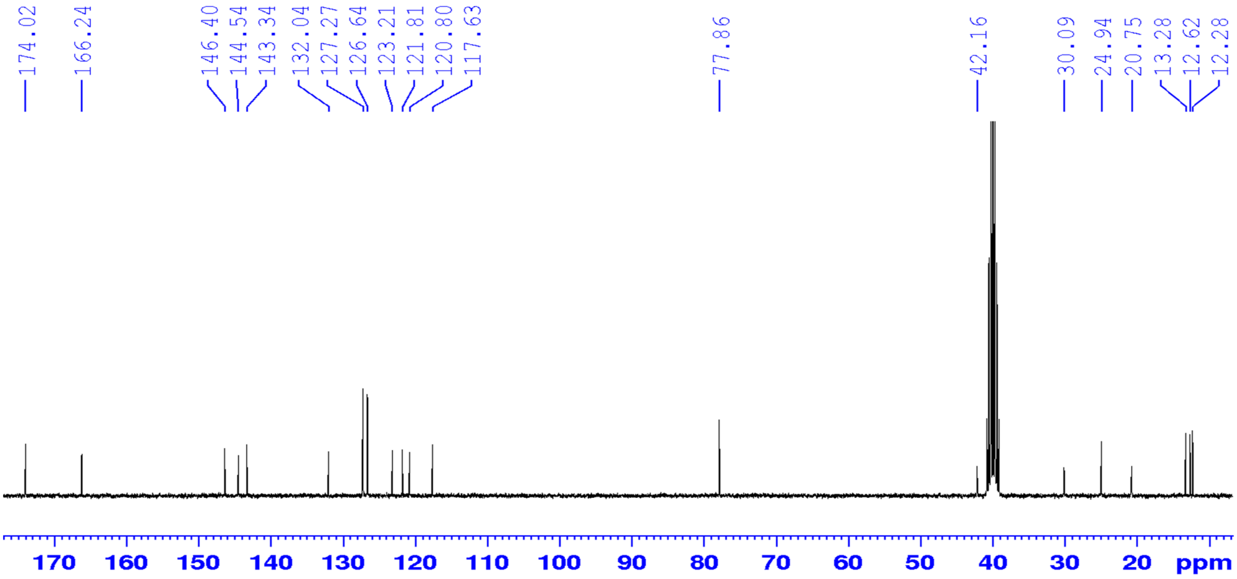
174.02
166.24
146.40
144.54
143.34
132.04
127.27
126.64
123.21
121.81
120.80
117.63
77.86
42.16
30.09
24.94
20.75
13.28
12.62
12.28
170 160 150 140 130 120 110 100 90 80 70 60 50 40 30 20 ppm

Compound #10

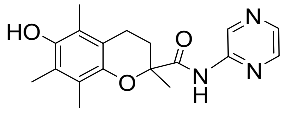
HO
O
N
H
N
N

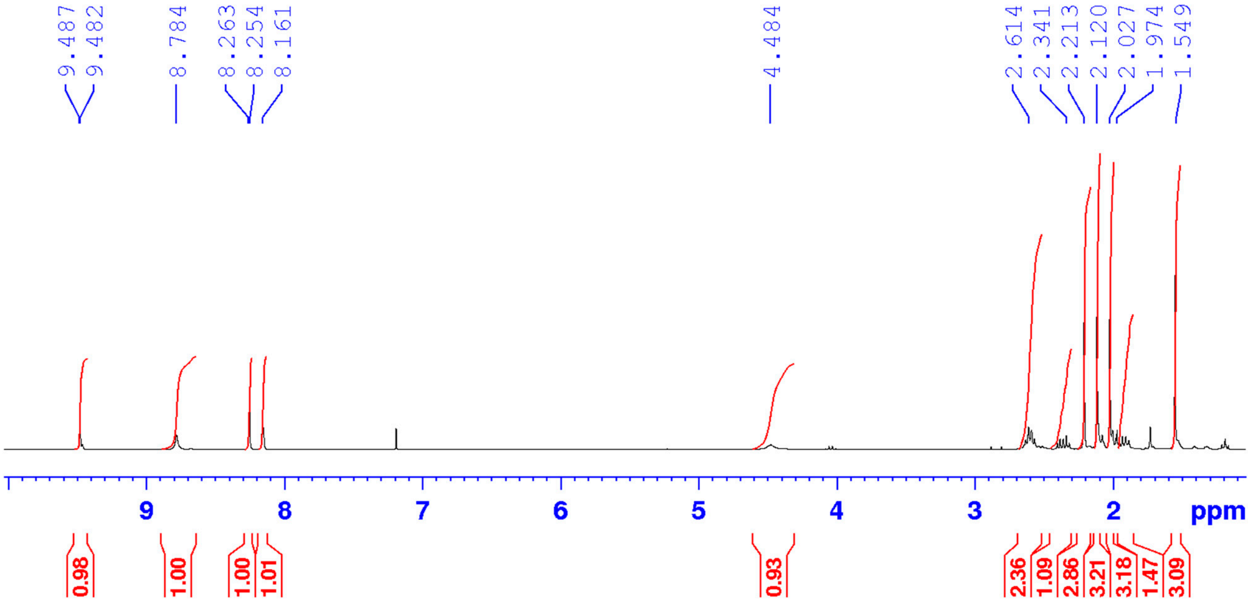
9.487
9.482
8.784
8.263
8.254
8.161
4.484
2.614
2.341
2.213
2.120
2.027
1.974
1.549
9
8
7
6
5
4
3
2
ppm
0.98
1.00
1.00
1.01
0.93
2.36
1.09
2.86
3.21
3.18
1.47
3.09

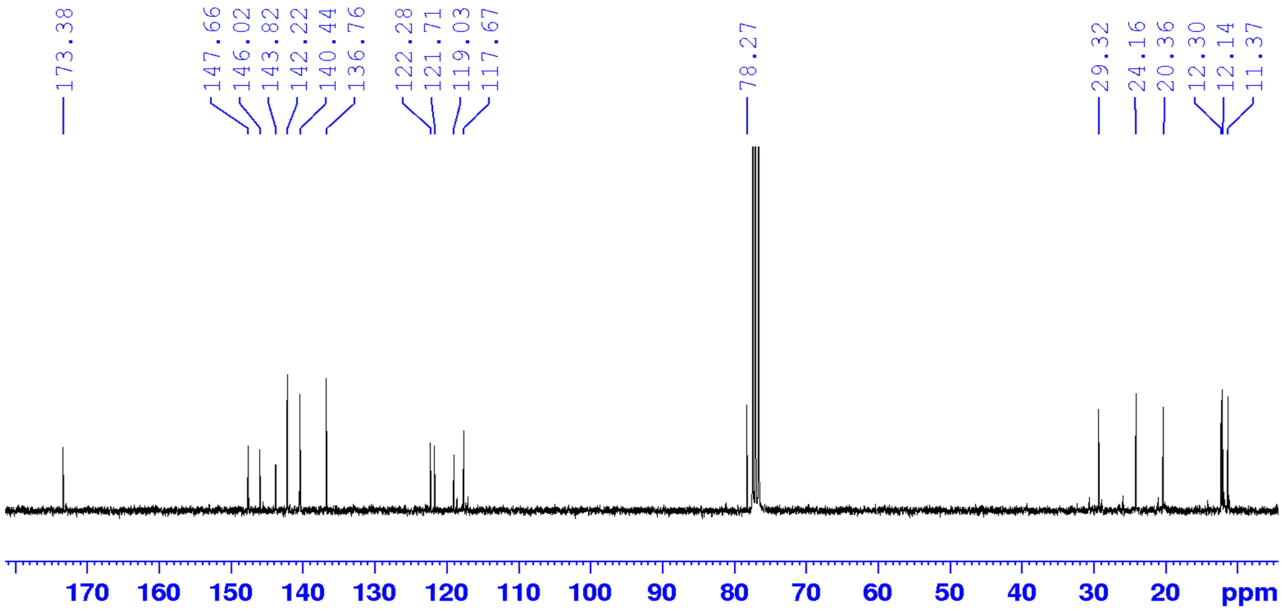
173.38
147.66
146.02
143.82
142.22
140.44
136.76
122.28
121.71
119.03
117.67
78.27
29.32
24.16
20.36
12.30
12.14
11.37
170
160
150
140
130
120
110
100
90
80
70
60
50
40
30
20
ppm

Compound #11

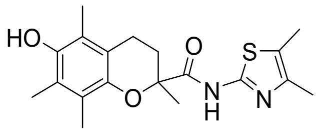
HO
O
O
N
H
S
N

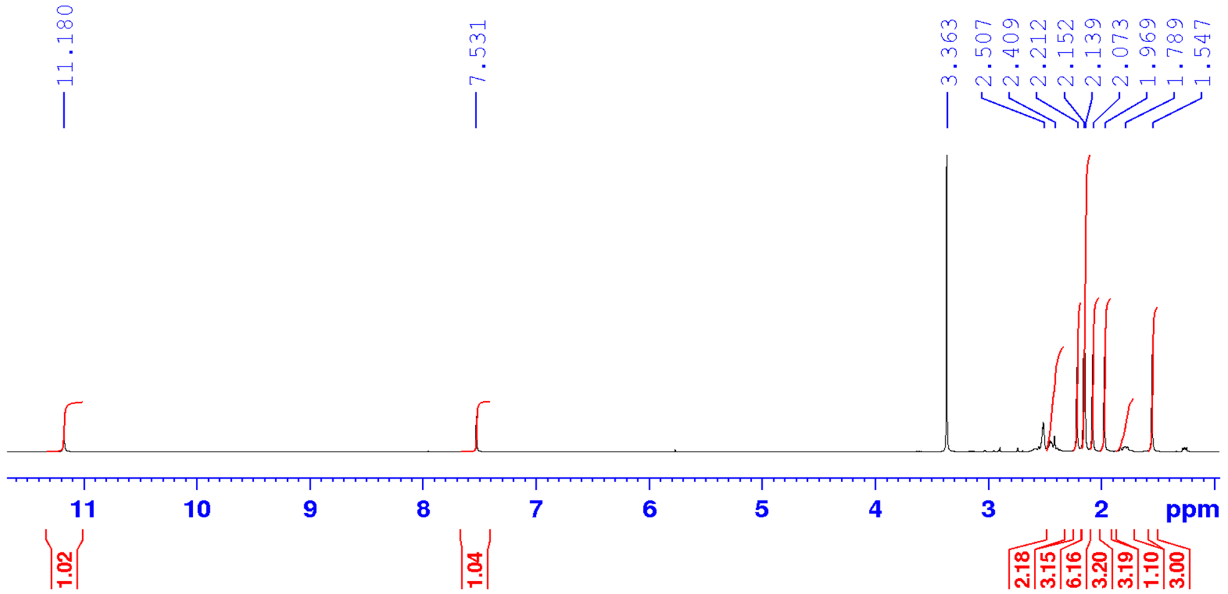
11.180
7.531
3.363
2.507
2.409
2.212
2.152
2.139
2.073
1.969
1.789
1.547
11
10
9
8
7
6
5
4
3
2
ppm
1.02
1.04
2.18
3.15
6.16
3.20
3.19
1.10
3.00

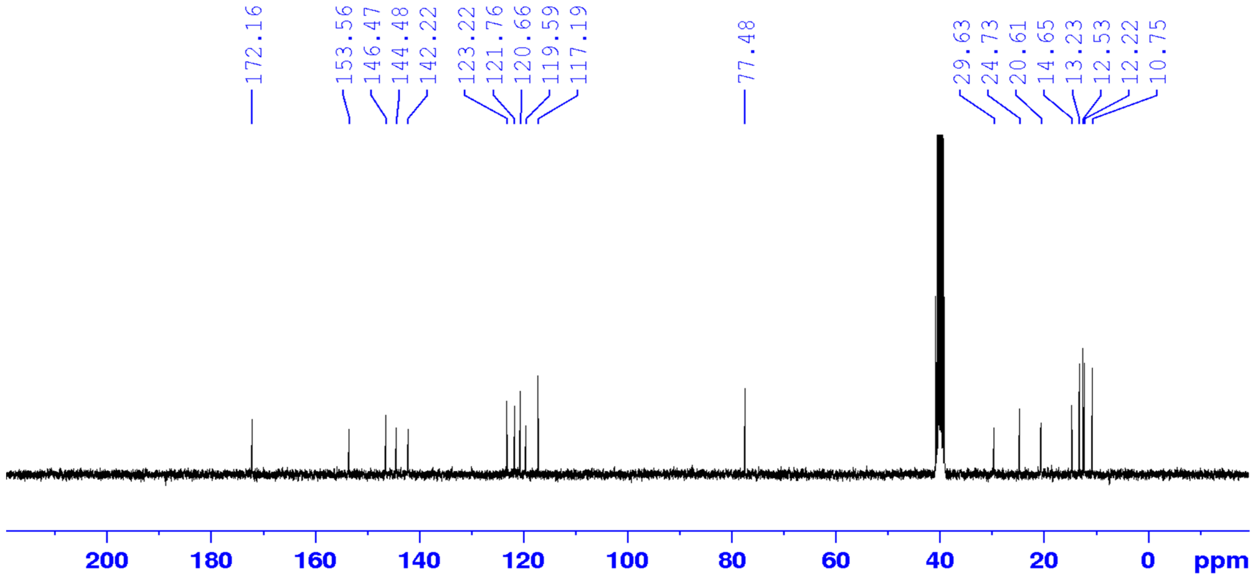
172.16
153.56
146.47
144.48
142.22
123.22
121.76
120.66
119.59
117.19
77.48
29.63
24.73
20.61
14.65
13.23
12.53
12.22
10.75
200
180
160
140
120
100
80
60
40
20
0
ppm

Compound #12

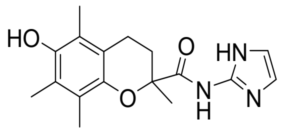

HO
O
HN
N
H
N
O

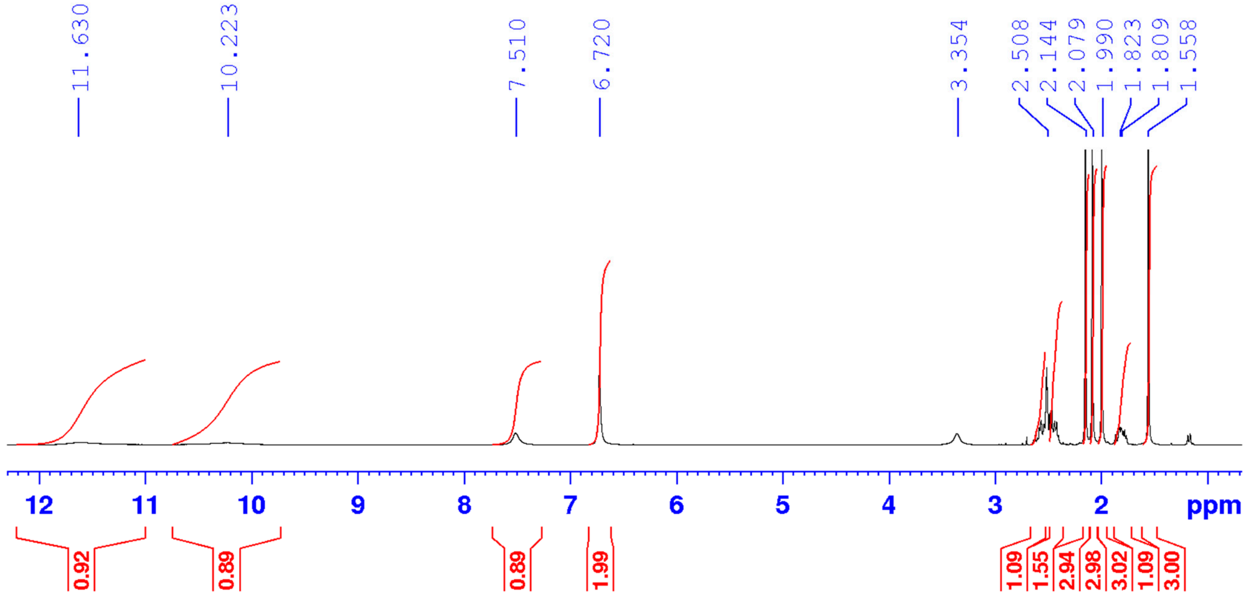

11.630
10.223
7.510
6.720
3.354
2.508
2.144
2.079
1.990
1.823
1.809
1.558
12
11
10
9
8
7
6
5
4
3
2
ppm
0.92
0.89
0.89
1.99
1.09
1.55
2.94
2.98
3.02
1.09
3.00

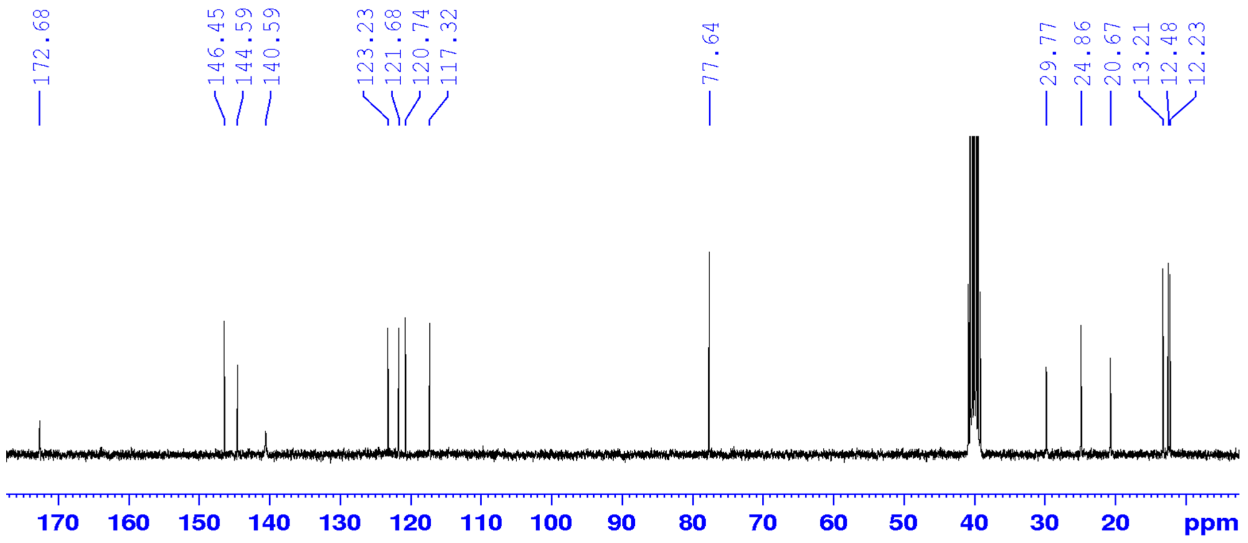

172.68
146.45
144.59
140.59
123.23
121.68
120.74
117.32
77.64
29.77
24.86
20.67
13.21
12.48
12.23
170
160
150
140
130
120
110
100
90
80
70
60
50
40
30
20
ppm

Compound #13

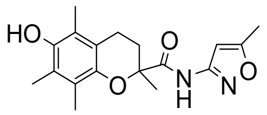
HO
O
N
H
N
O

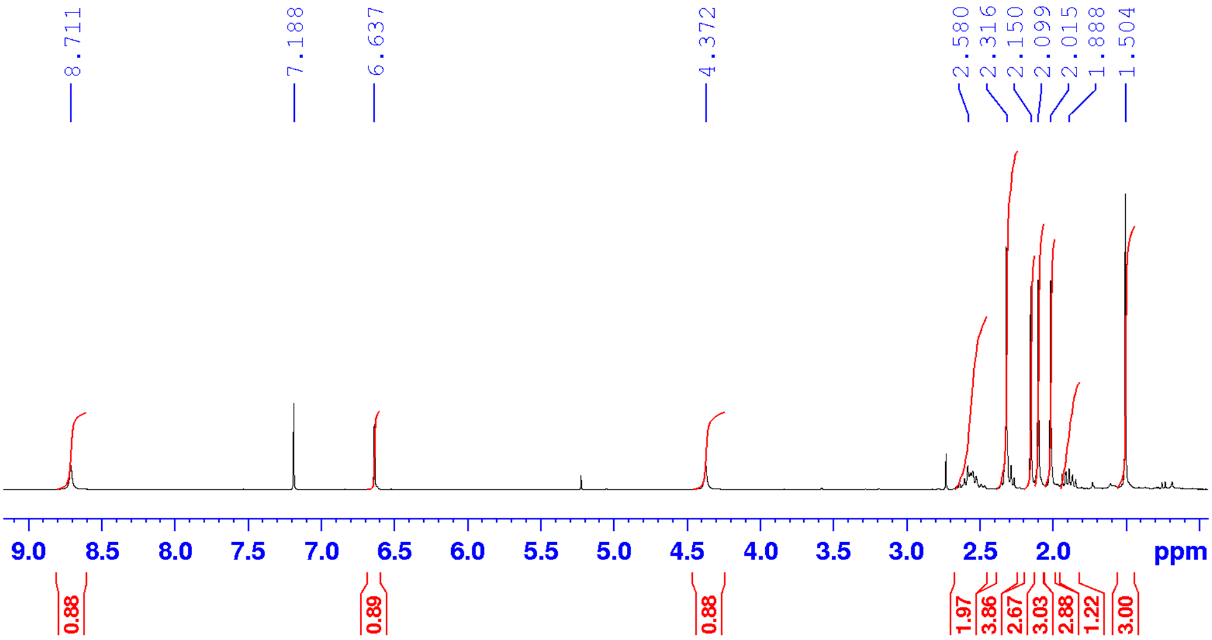
8.711
7.188
6.637
4.372
2.580
2.316
2.150
2.099
2.015
1.888
1.504
9.0 8.5 8.0 7.5 7.0 6.5 6.0 5.5 5.0 4.5 4.0 3.5 3.0 2.5 2.0 ppm
0.88
0.89
0.88
1.97
3.86
2.67
3.03
2.88
1.22
3.00

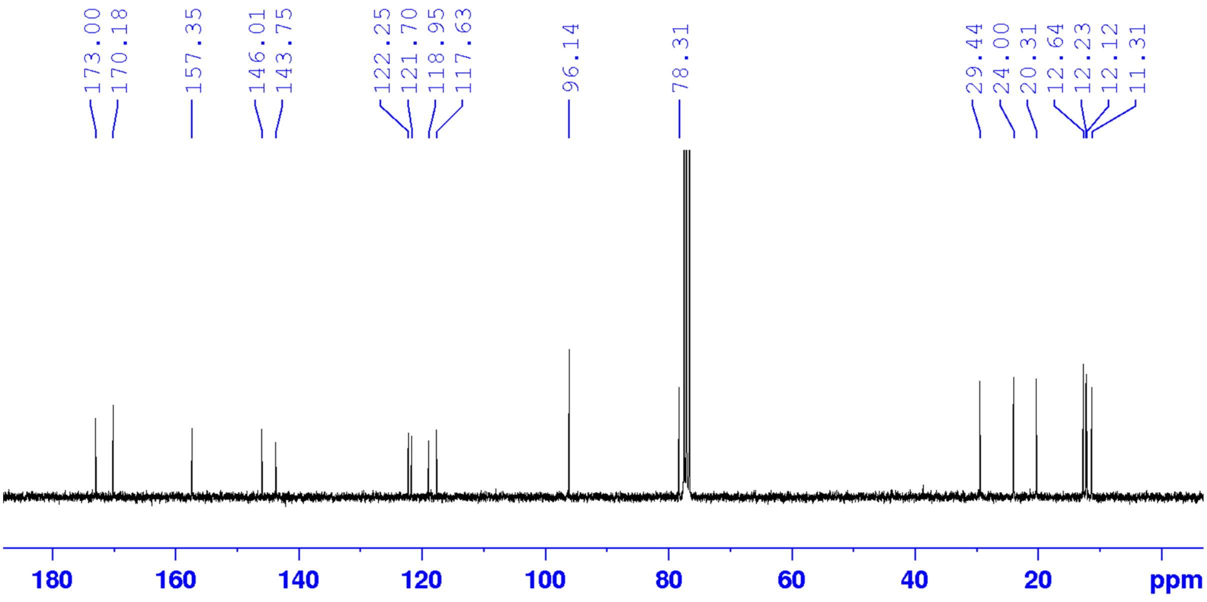
173.00
170.18
157.35
146.01
143.75
122.25
121.70
118.95
117.63
96.14
78.31
29.44
24.00
20.31
12.64
12.23
12.12
11.31
180 160 140 120 100 80 60 40 20 ppm

Compound #14

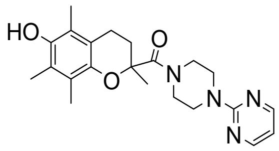
HO
O
N
N
N
N
O

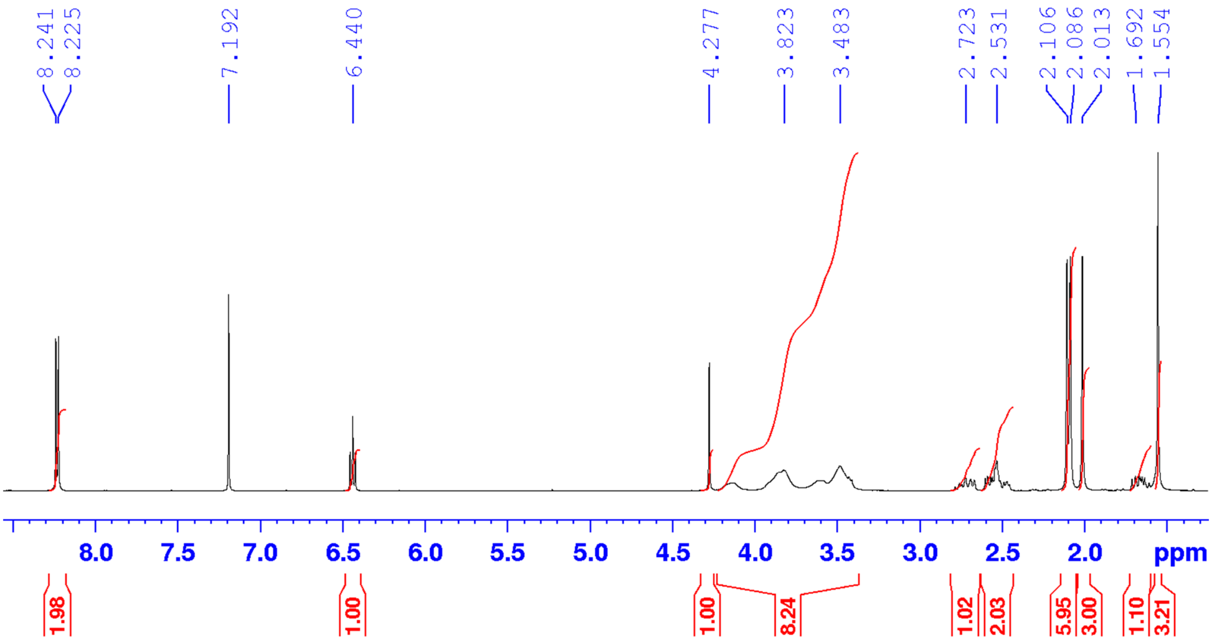
8.241
8.225
7.192
6.440
4.277
3.823
3.483
2.723
2.531
2.106
2.086
2.013
1.692
1.554
8.0 7.5 7.0 6.5 6.0 5.5 5.0 4.5 4.0 3.5 3.0 2.5 2.0 ppm
1.98
1.00
1.00
8.24
1.02
2.03
5.95
3.00
1.10
3.21

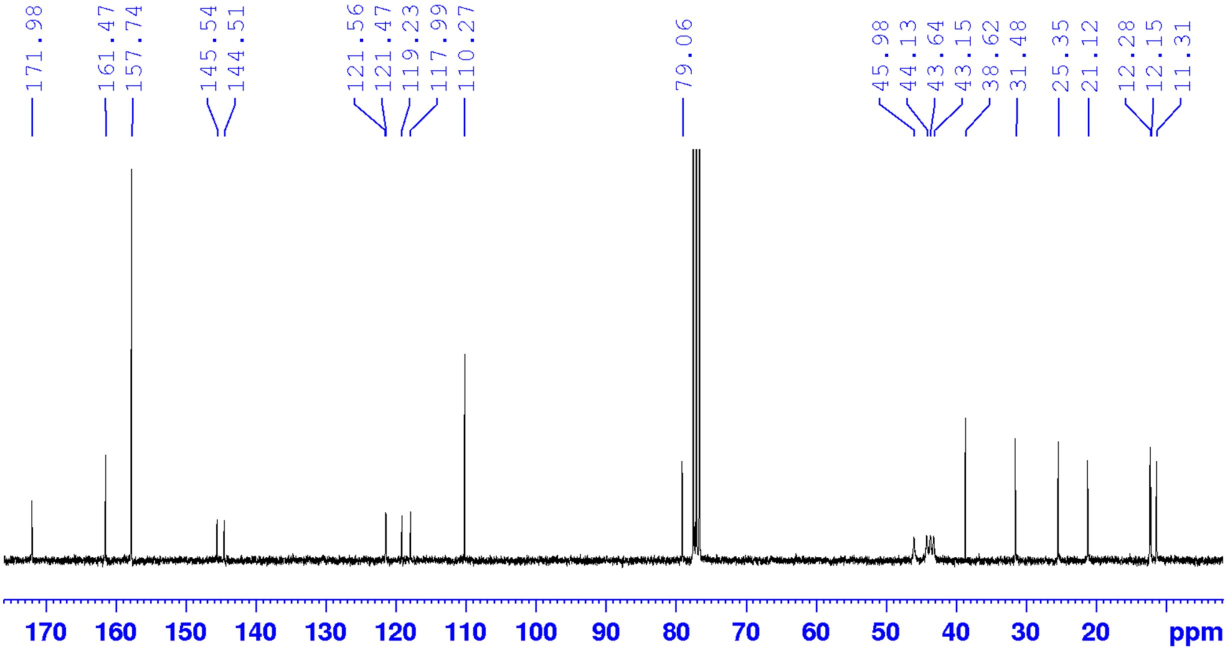
171.98
161.47
157.74
145.54
144.51
121.56
121.47
119.23
117.99
110.27
79.06
45.98
44.13
43.64
43.15
38.62
31.48
25.35
21.12
12.28
12.15
11.31
170 160 150 140 130 120 110 100 90 80 70 60 50 40 30 20 ppm

Compound #15

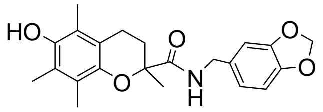
HO
O
N
H
O
O
O

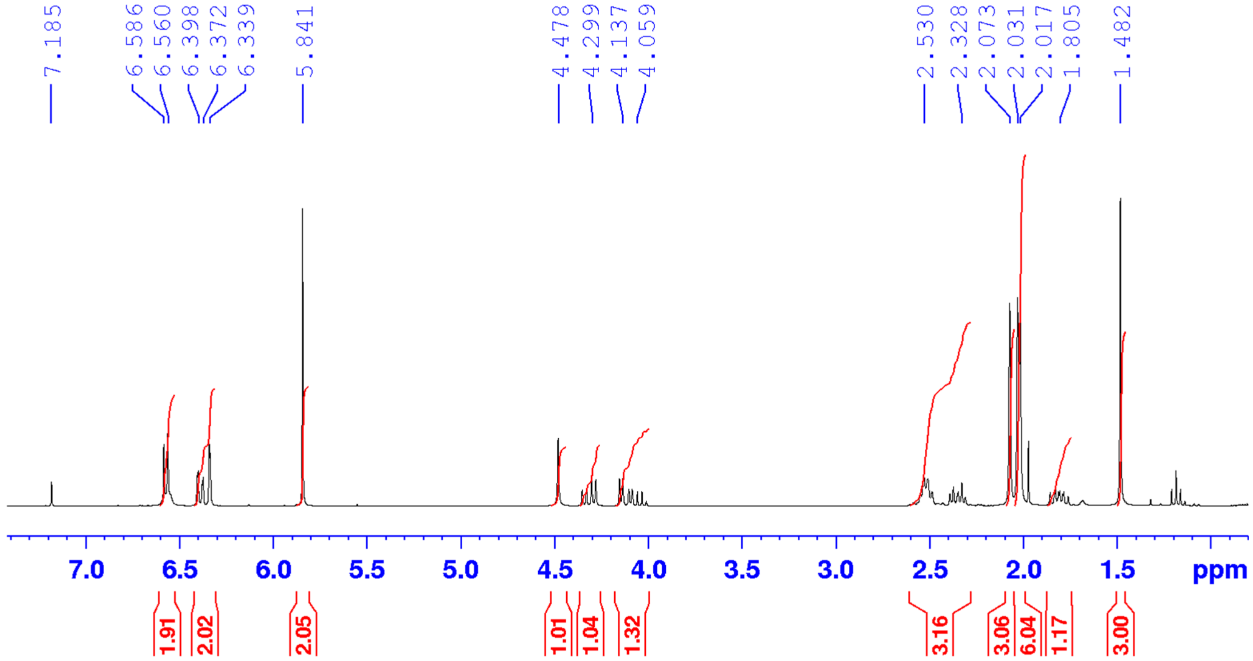
7.185
6.586
6.560
6.398
6.372
6.339
5.841
4.478
4.299
4.137
4.059
2.530
2.328
2.073
2.031
2.017
1.805
1.482
7.0 6.5 6.0 5.5 5.0 4.5 4.0 3.5 3.0 2.5 2.0 1.5 ppm
1.91
2.02
2.05
1.01
1.04
1.32
3.16
3.06
6.04
1.17
3.00

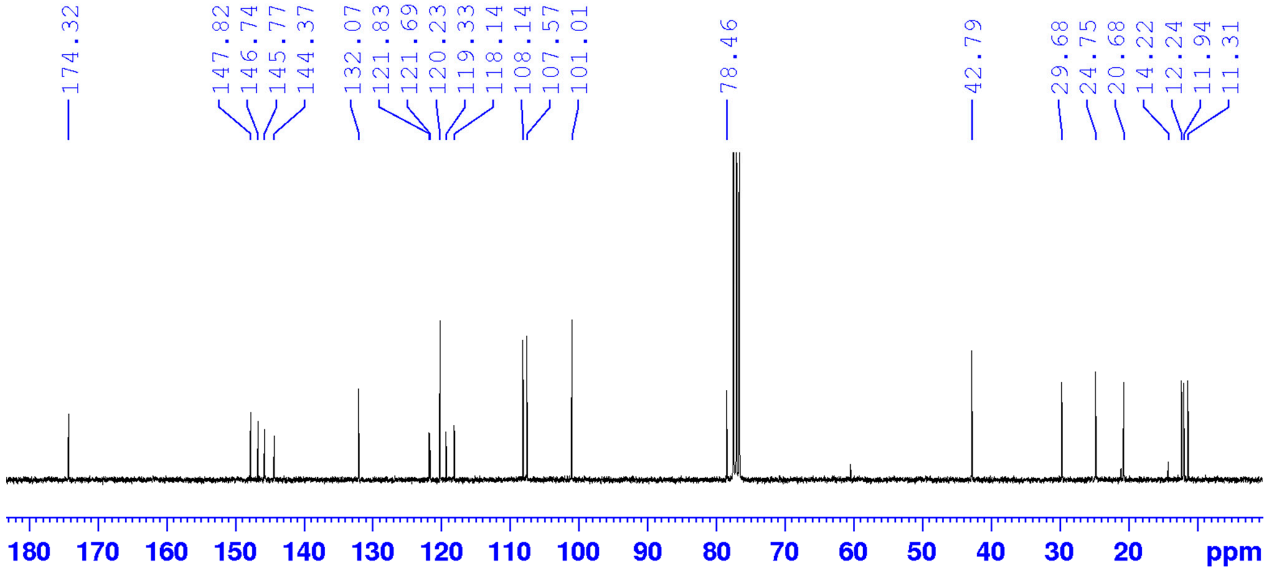
174.32
147.82
146.74
145.77
144.37
132.07
121.83
121.69
120.23
119.33
118.14
108.14
107.57
101.01
78.46
42.79
29.68
24.75
20.68
14.22
12.24
11.94
11.31
180 170 160 150 140 130 120 110 100 90 80 70 60 50 40 30 20 ppm

Compound #16

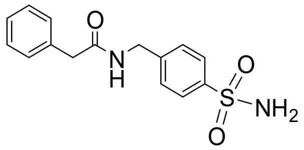
O
N
H
O
S
NH$_2$
O

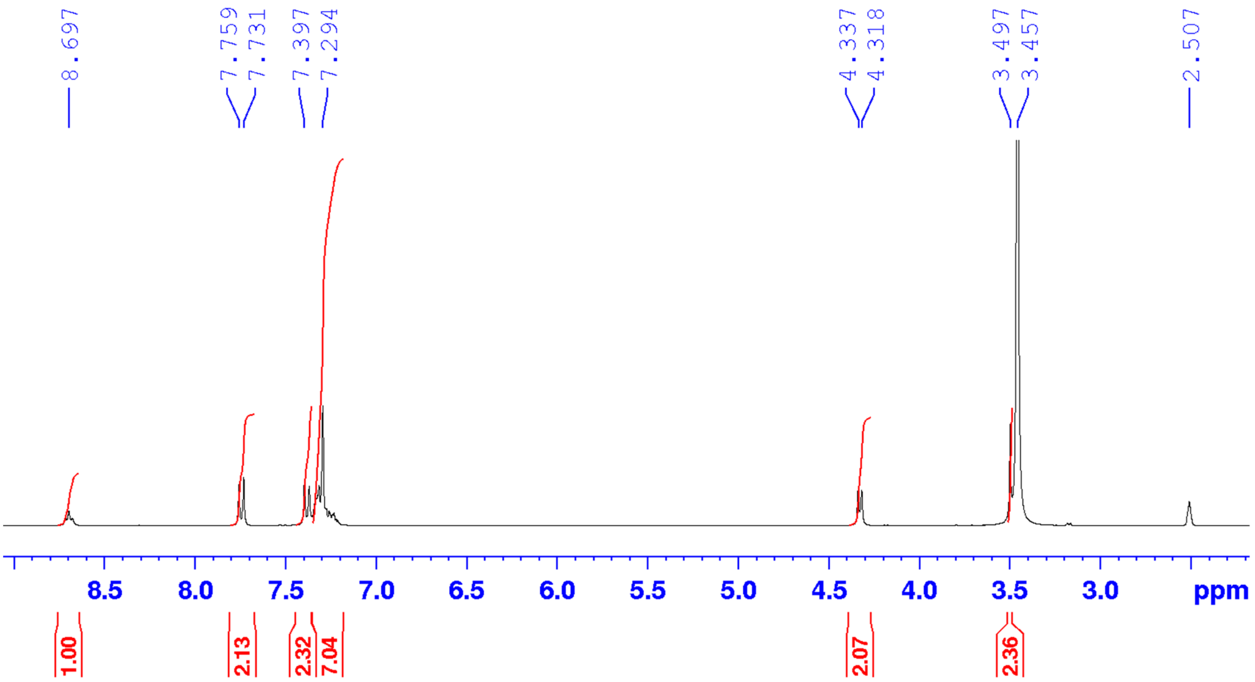
8.697
7.759
7.731
7.397
7.294
4.337
4.318
3.497
3.457
2.507
8.5
8.0
7.5
7.0
6.5
6.0
5.5
5.0
4.5
4.0
3.5
3.0
ppm
1.00
2.13
2.32
7.04
2.07
2.36

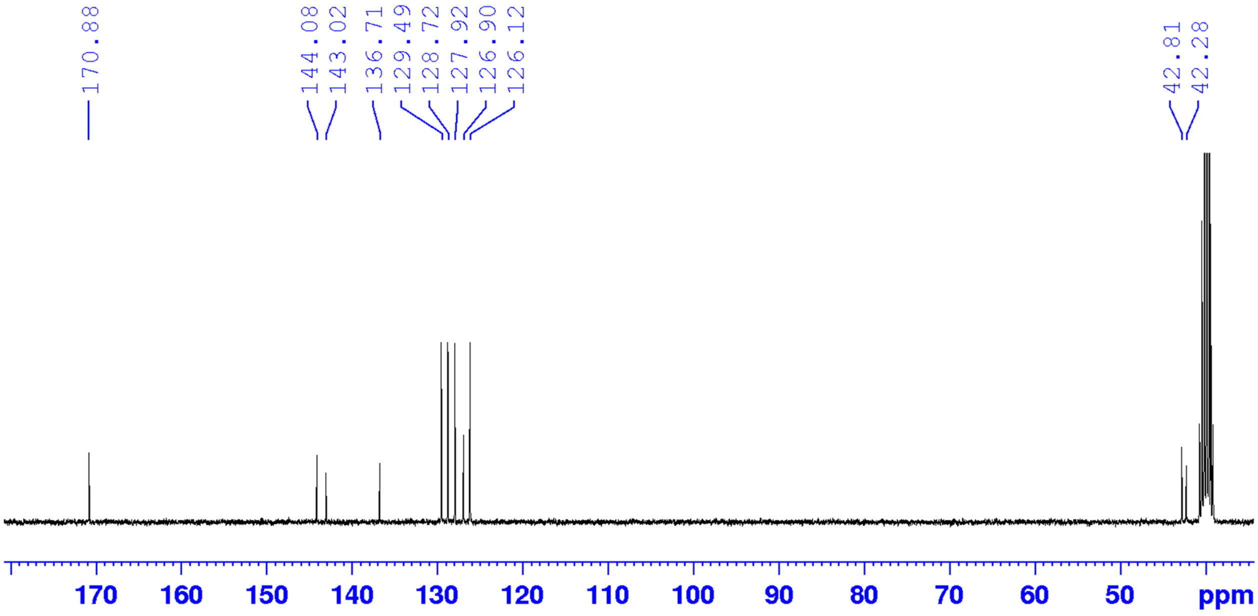
170.88
144.08
143.02
136.71
129.49
128.72
127.92
126.90
126.12
42.81
42.28
170
160
150
140
130
120
110
100
90
80
70
60
50
ppm

Compound #17

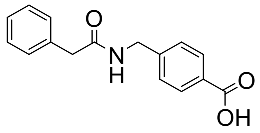
O
N
H
O
OH

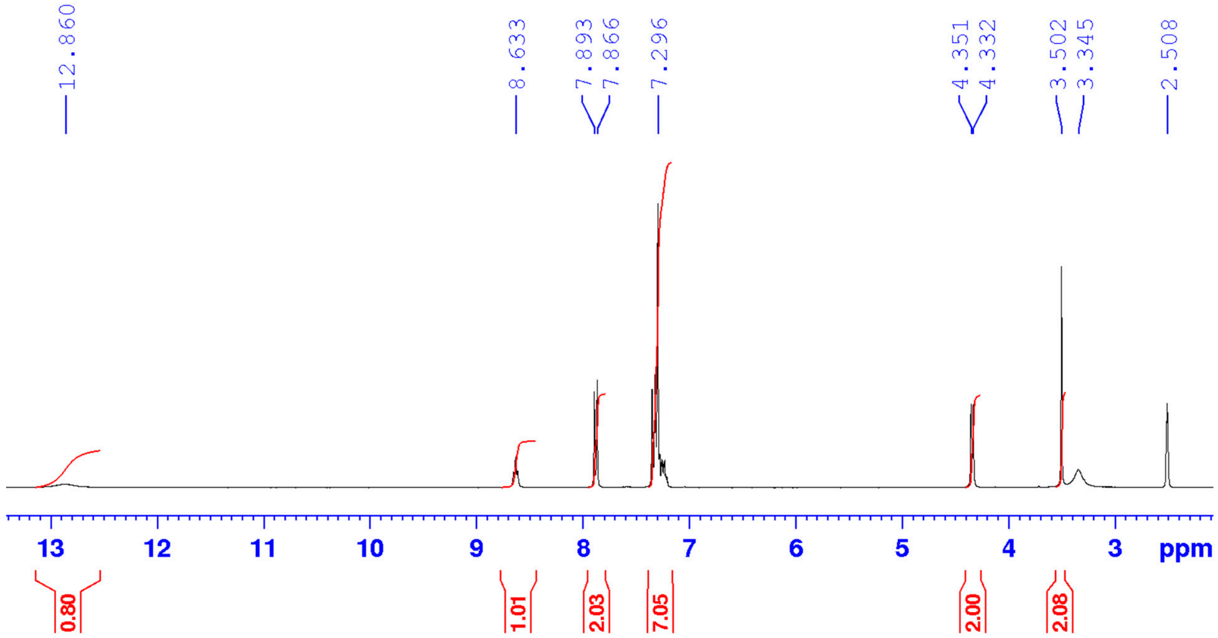
12.860
8.633
7.893
7.866
7.296
4.351
4.332
3.502
3.345
2.508
13
12
11
10
9
8
7
6
5
4
3
ppm
0.80
1.01
2.03
7.05
2.00
2.08

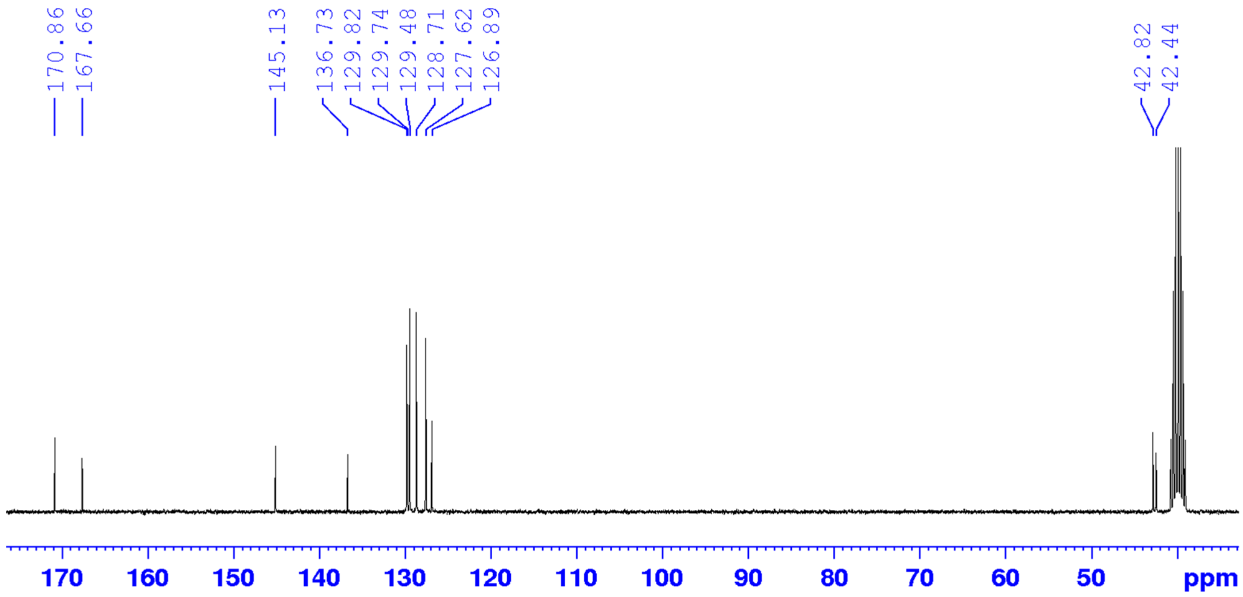
170.86
167.66
145.13
136.73
129.82
129.74
129.48
128.71
127.62
126.89
42.82
42.44
170
160
150
140
130
120
110
100
90
80
70
60
50
ppm

Compound #18

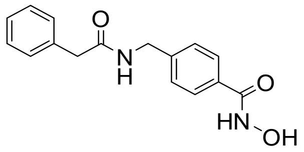
O
N
H
O
HN
OH

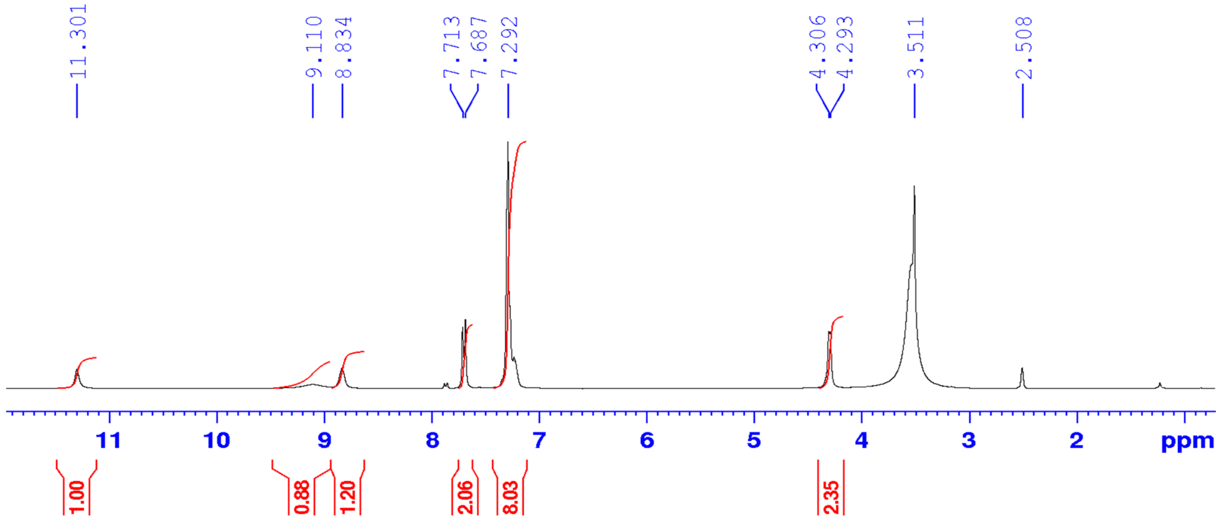
11.301
9.110
8.834
7.713
7.687
7.292
4.306
4.293
3.511
2.508
11
10
9
8
7
6
5
4
3
2
ppm
1.00
0.88
1.20
2.06
8.03
2.35

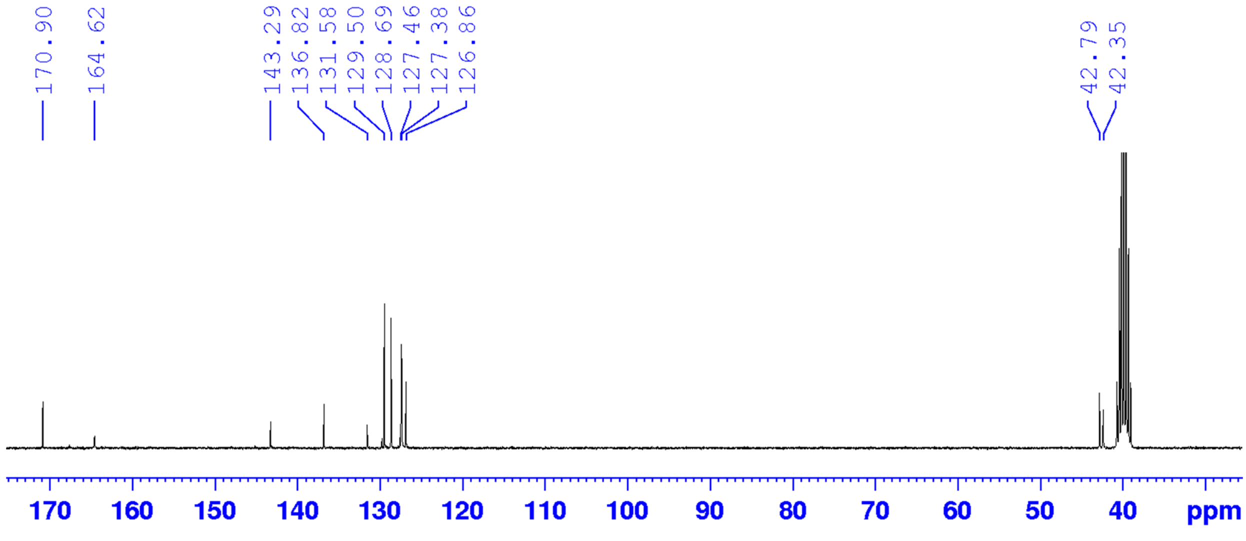
170.90
164.62
143.29
136.82
131.58
129.50
128.69
127.46
127.38
126.86
42.79
42.35
170
160
150
140
130
120
110
100
90
80
70
60
50
40
ppm

Compound #19

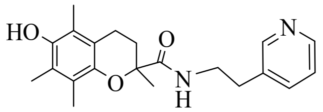
HO
O
N
O
N
H

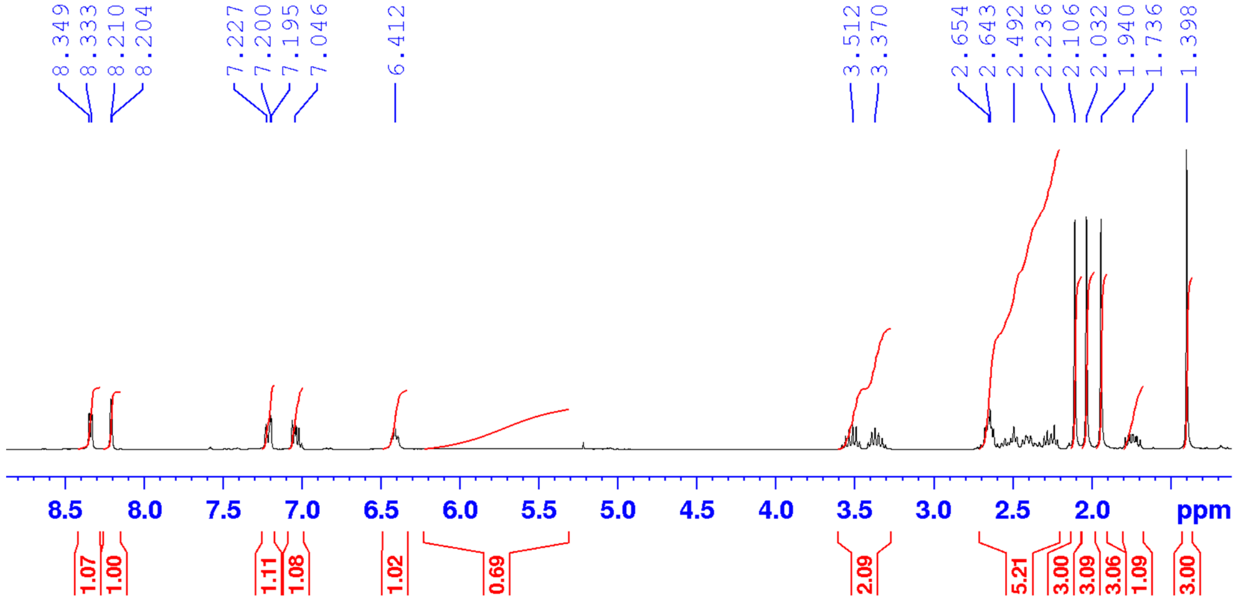
8.349
8.333
8.210
8.204
7.227
7.200
7.195
7.046
6.412
3.512
3.370
2.654
2.643
2.492
2.236
2.106
2.032
1.940
1.736
1.398
8.5
8.0
7.5
7.0
6.5
6.0
5.5
5.0
4.5
4.0
3.5
3.0
2.5
2.0
ppm
1.07
1.00
1.11
1.08
1.02
0.69
2.09
5.21
3.00
3.09
3.06
1.09
3.00

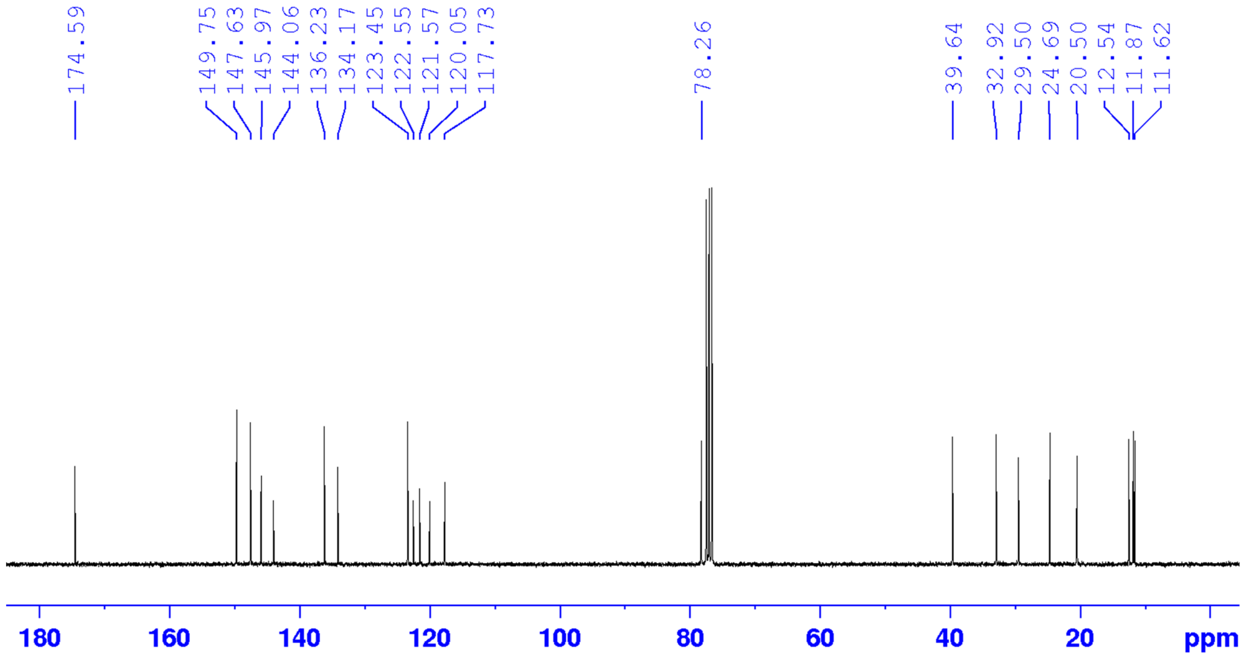
174.59
149.75
147.63
145.97
144.06
136.23
134.17
123.45
122.55
121.57
120.05
117.73
78.26
39.64
32.92
29.50
24.69
20.50
12.54
11.87
11.62
180
160
140
120
100
80
60
40
20
ppm

Compound #20

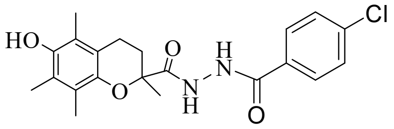
HO
O
O
H
N
N
H
O
Cl

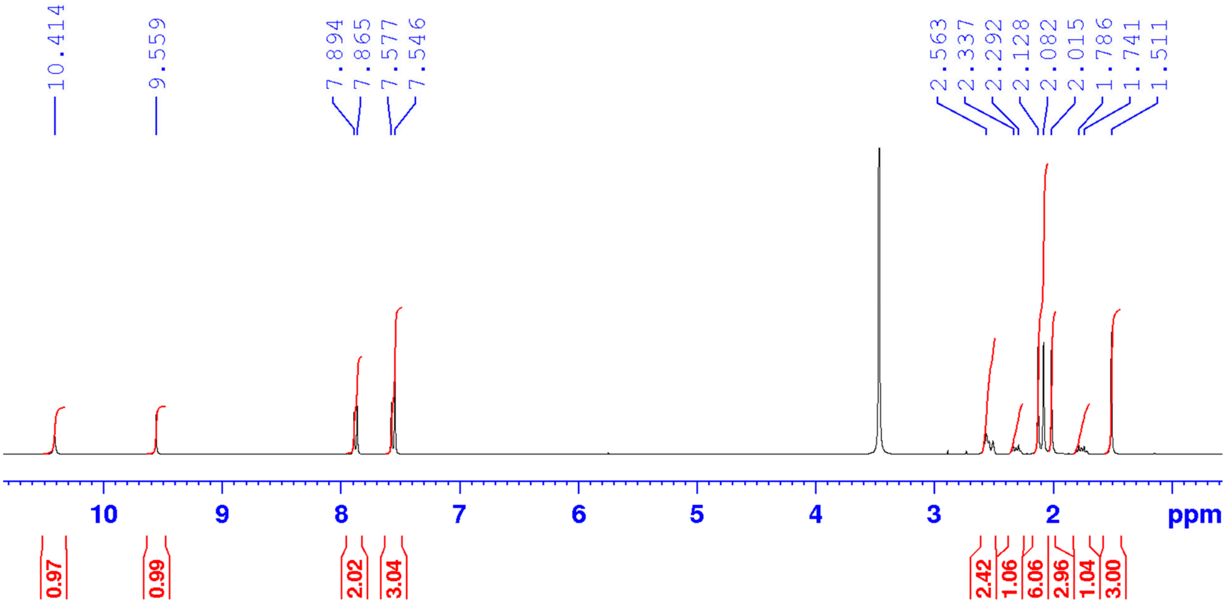
10.414
9.559
7.894
7.865
7.577
7.546
2.563
2.337
2.292
2.128
2.082
2.015
1.786
1.741
1.511
10 9 8 7 6 5 4 3 2 ppm
0.97
0.99
2.02
3.04
2.42
1.06
6.06
2.96
1.04
3.00

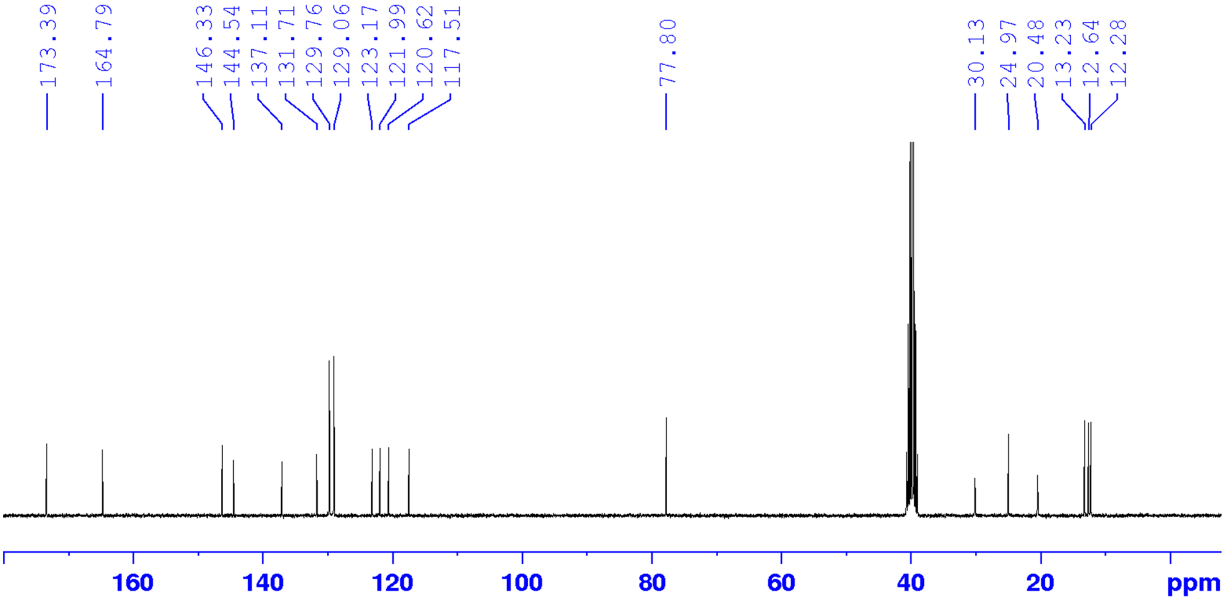
173.39
164.79
146.33
144.54
137.11
131.71
129.76
129.06
123.17
121.99
120.62
117.51
77.80
30.13
24.97
20.48
13.23
12.64
12.28
160 140 120 100 80 60 40 20 ppm
