# Supplementary material for: Phenylacetyl-/Trolox- Amides: Synthesis, Sigma-1, HDAC-6, and Antioxidant Activities
Source: Int J Mol Sci. 2023 Oct 18;24(20):15295. doi: 10.3390/ijms242015295 (PMC10607876; doi:10.3390/ijms242015295)
Supplement: Supplementary file 1 [file ijms-24-15295-s001.zip › Supplemental ORAC Plots.pdf]

*ORAC Standard Curves [averaged slopes (n=3 runs) are reported in **table 5** and illustrated in **figure 4**] of the manuscript.*

- Trolox Standard Curves.

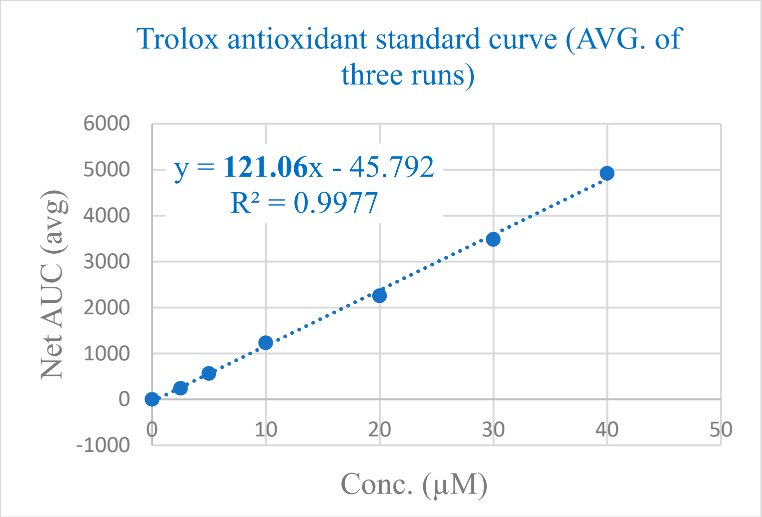

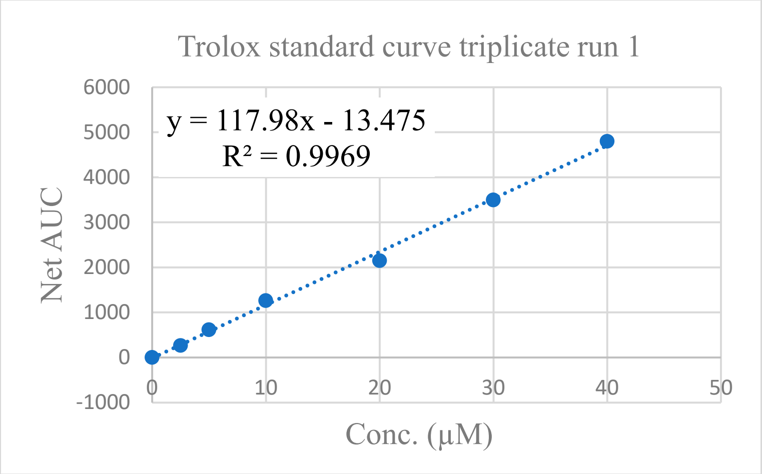

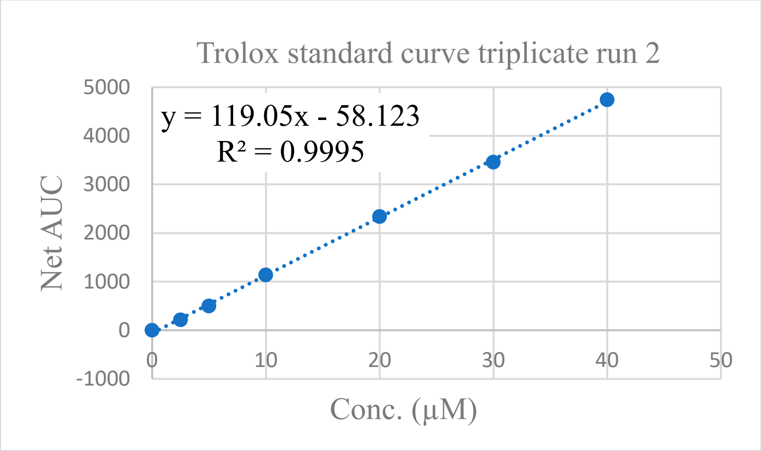

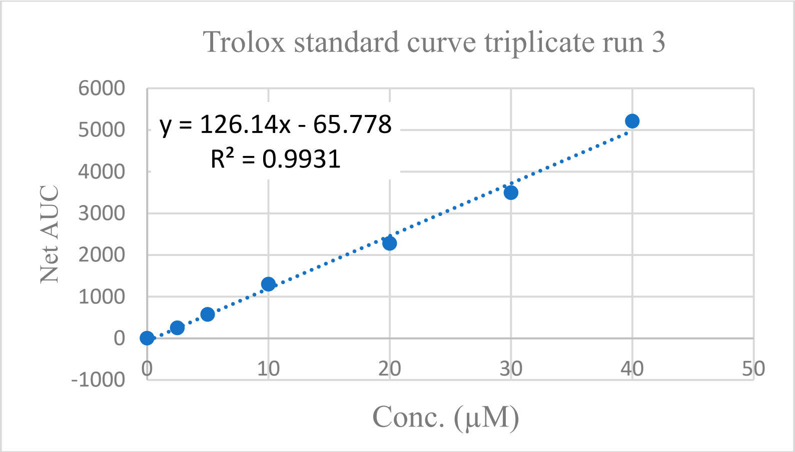

- Test Compounds (**3, 4, 8, 12, 18, 20**) Standard Curves.

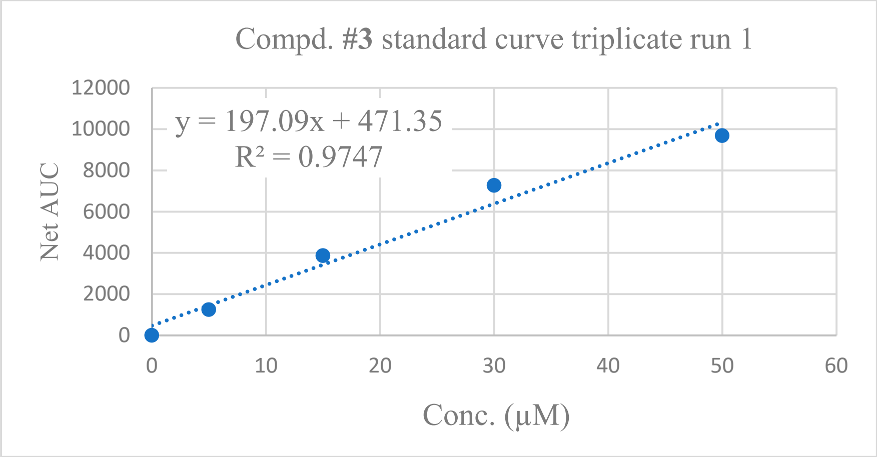

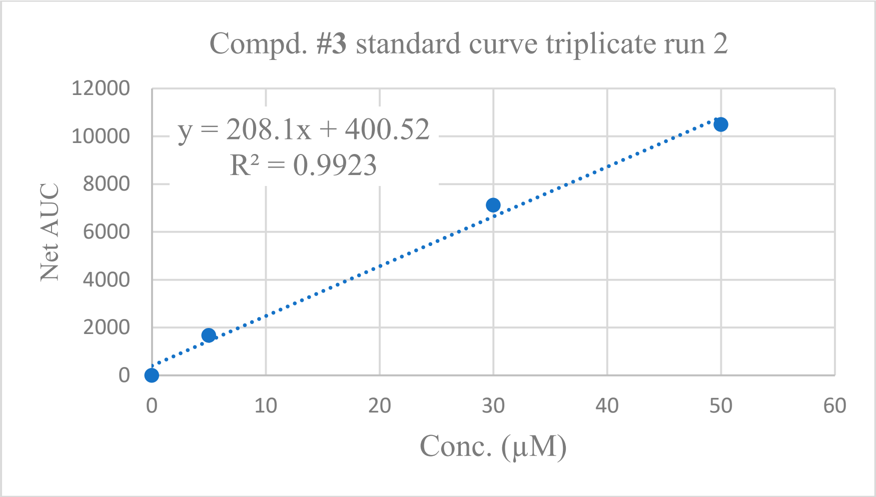

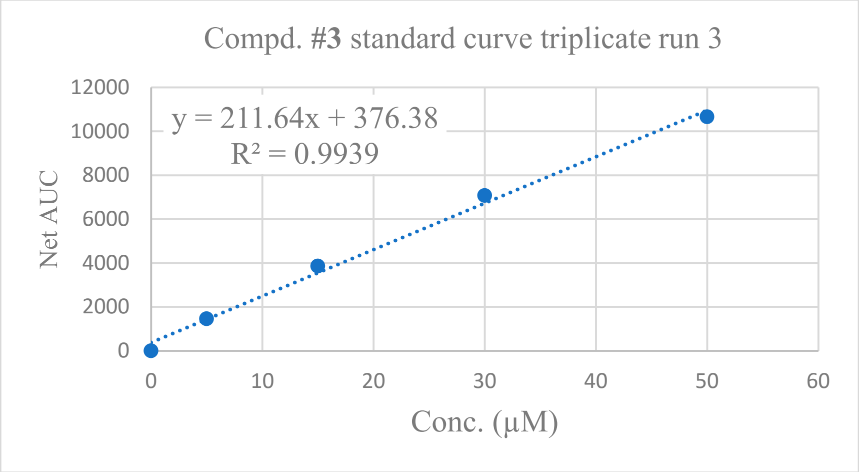
Compd. #3 standard curve triplicate run 3
y = 211.64x + 376.38
R² = 0.9939
Net AUC
Conc. (µM)
0
2000
4000
6000
8000
10000
12000
0
10
20
30
40
50
60

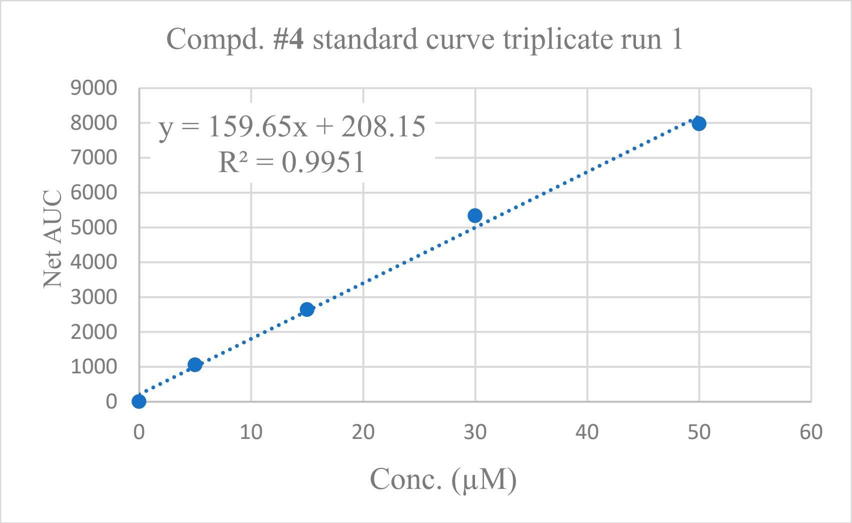
Compd. #4 standard curve triplicate run 1
y = 159.65x + 208.15
R² = 0.9951
Net AUC
Conc. (µM)
0
1000
2000
3000
4000
5000
6000
7000
8000
9000
0
10
20
30
40
50
60

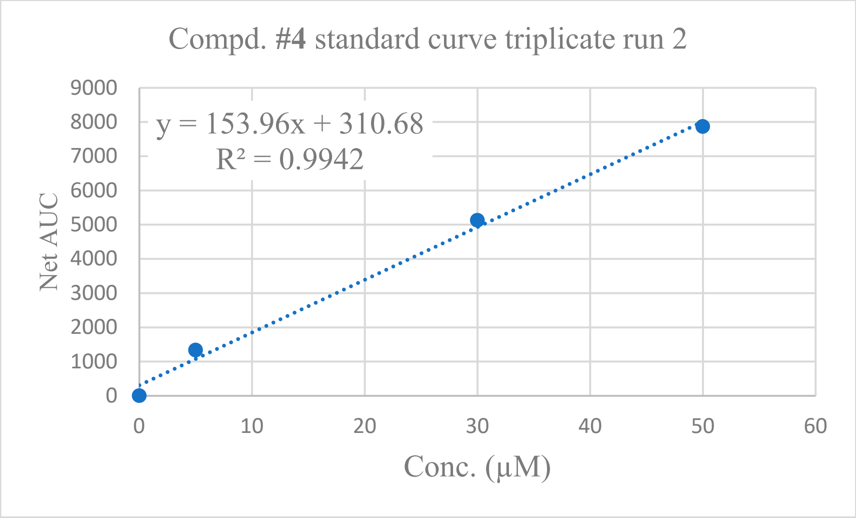
Compd. #4 standard curve triplicate run 2
y = 153.96x + 310.68
R² = 0.9942
Net AUC
Conc. (µM)
0
1000
2000
3000
4000
5000
6000
7000
8000
9000
0
10
20
30
40
50
60

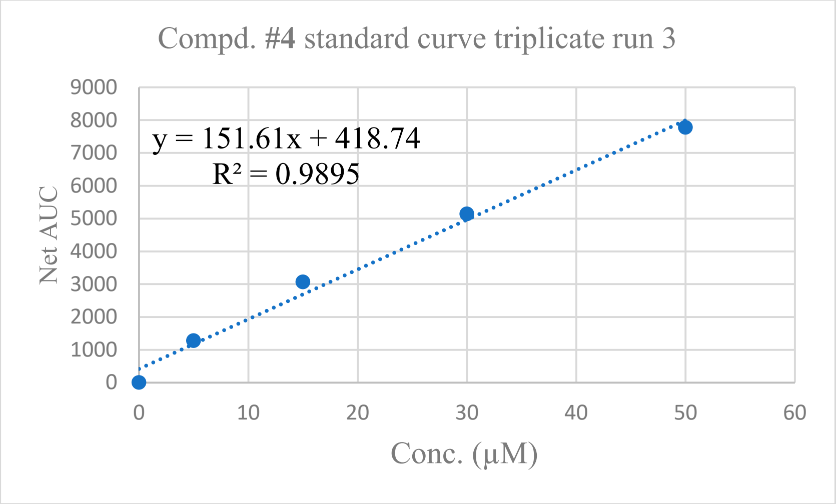
Compd. #4 standard curve triplicate run 3
y = 151.61x + 418.74
R² = 0.9895
Net AUC
Conc. (µM)
9000
8000
7000
6000
5000
4000
3000
2000
1000
0
0
10
20
30
40
50
60

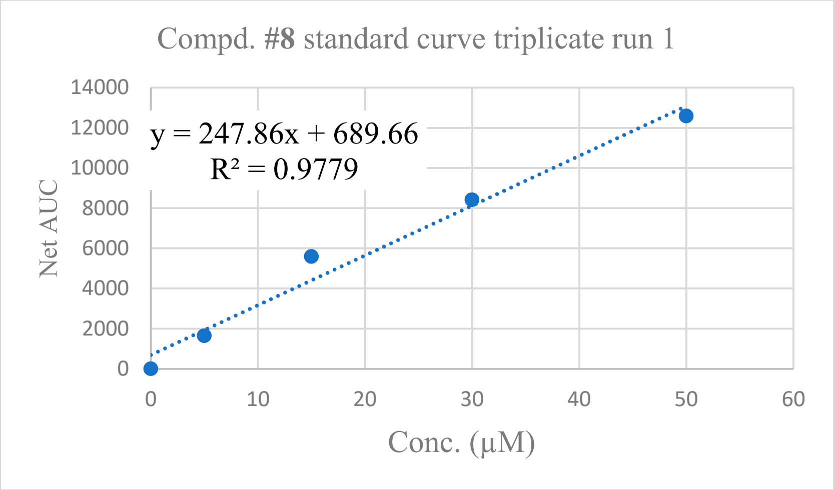
Compd. #8 standard curve triplicate run 1
y = 247.86x + 689.66
R² = 0.9779
Net AUC
Conc. (µM)
14000
12000
10000
8000
6000
4000
2000
0
0
10
20
30
40
50
60

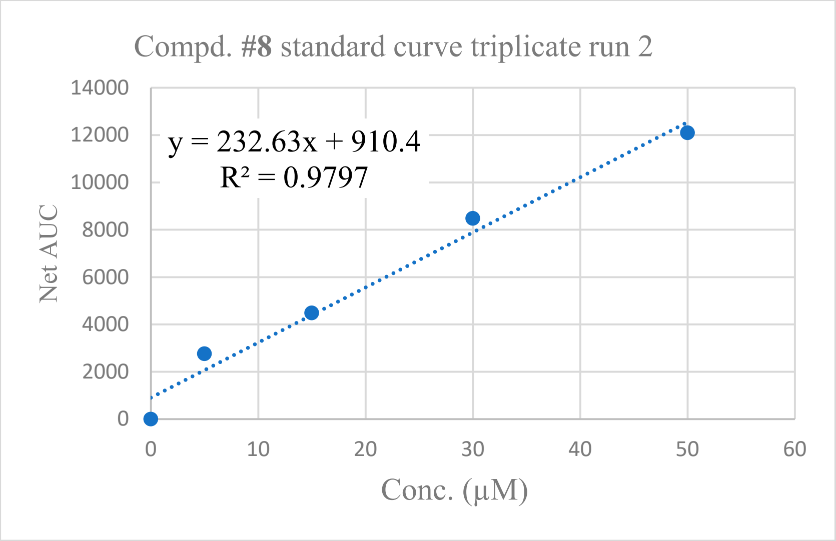
Compd. #8 standard curve triplicate run 2
y = 232.63x + 910.4
R² = 0.9797
Net AUC
Conc. (µM)
14000
12000
10000
8000
6000
4000
2000
0
0
10
20
30
40
50
60

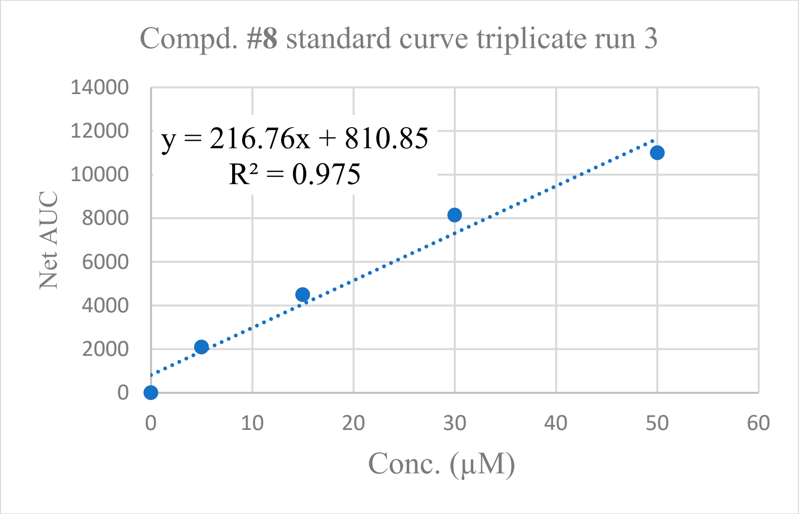
Compd. #8 standard curve triplicate run 3
y = 216.76x + 810.85
R² = 0.975
Net AUC
Conc. (µM)
14000
12000
10000
8000
6000
4000
2000
0
0
10
20
30
40
50
60

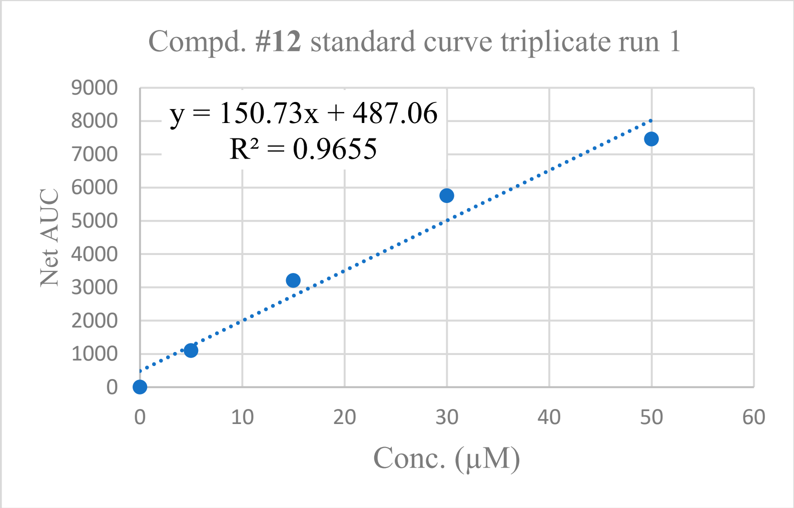
Compd. #12 standard curve triplicate run 1
y = 150.73x + 487.06
R² = 0.9655
Net AUC
Conc. (µM)
9000
8000
7000
6000
5000
4000
3000
2000
1000
0
0
10
20
30
40
50
60

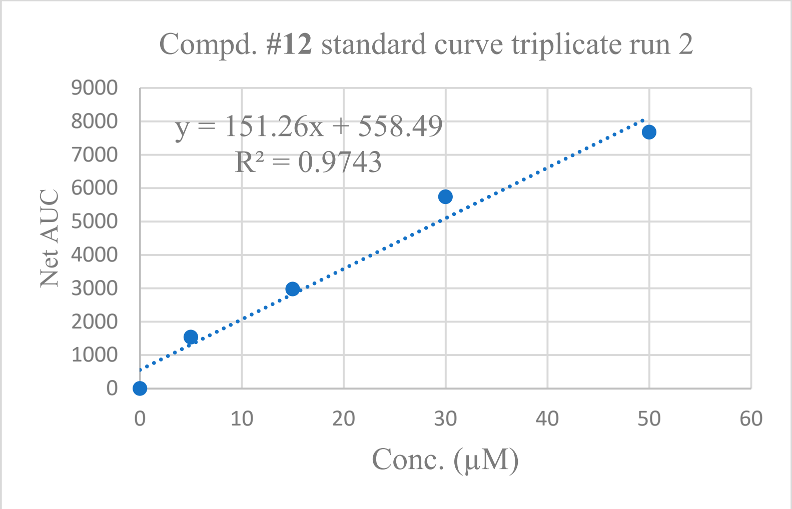
Compd. #12 standard curve triplicate run 2
y = 151.26x + 558.49
R² = 0.9743
Net AUC
Conc. (µM)
9000
8000
7000
6000
5000
4000
3000
2000
1000
0
0
10
20
30
40
50
60

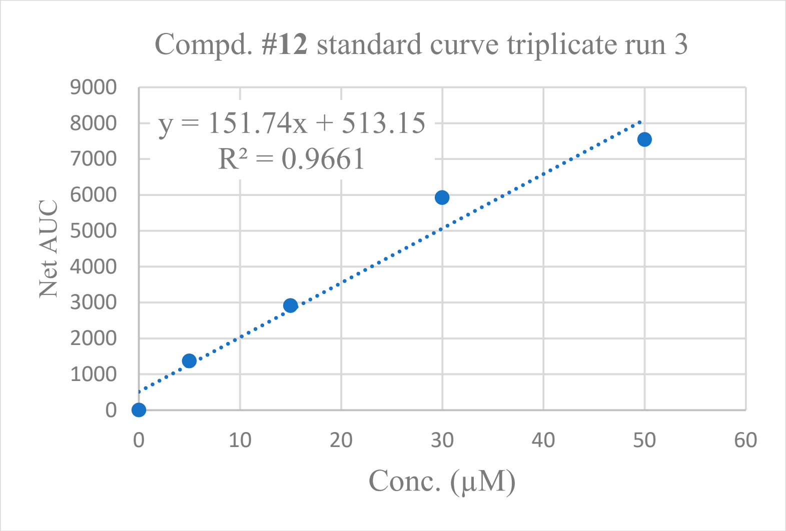

Compd. #12 standard curve triplicate run 3
y = 151.74x + 513.15
R² = 0.9661
Net AUC
Conc. (μM)
0
1000
2000
3000
4000
5000
6000
7000
8000
9000
0
10
20
30
40
50
60

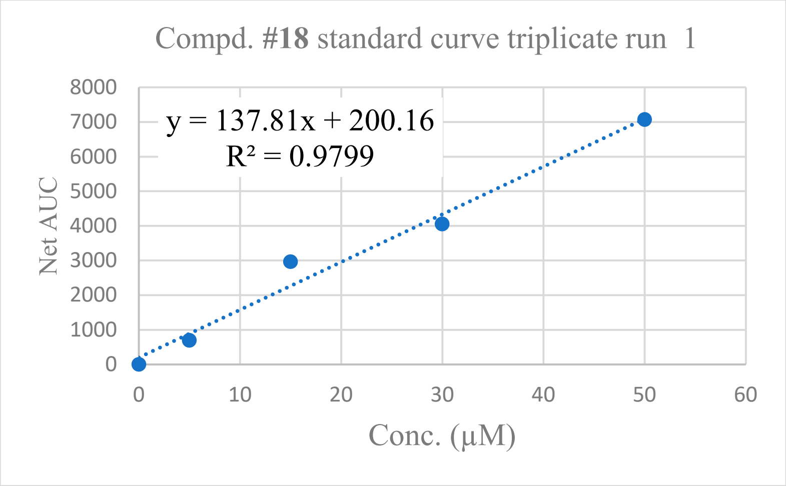

Compd. #18 standard curve triplicate run 1
y = 137.81x + 200.16
R² = 0.9799
Net AUC
Conc. (μM)
0
1000
2000
3000
4000
5000
6000
7000
8000
0
10
20
30
40
50
60

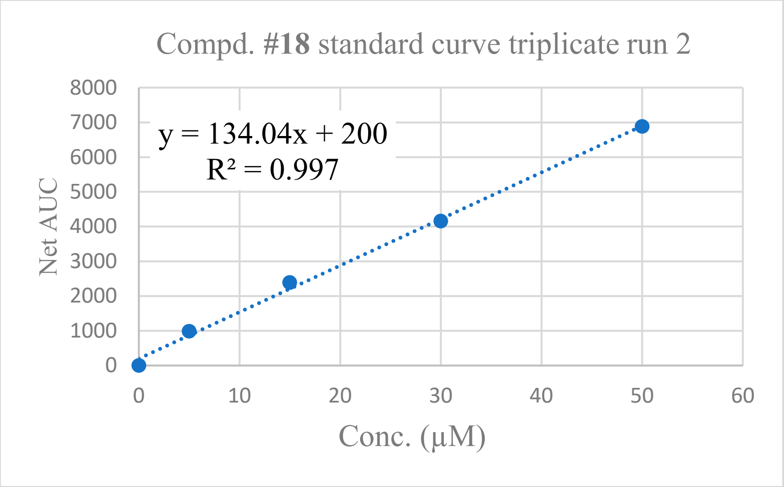

Compd. #18 standard curve triplicate run 2
y = 134.04x + 200
R² = 0.997
Net AUC
Conc. (μM)
0
1000
2000
3000
4000
5000
6000
7000
8000
0
10
20
30
40
50
60

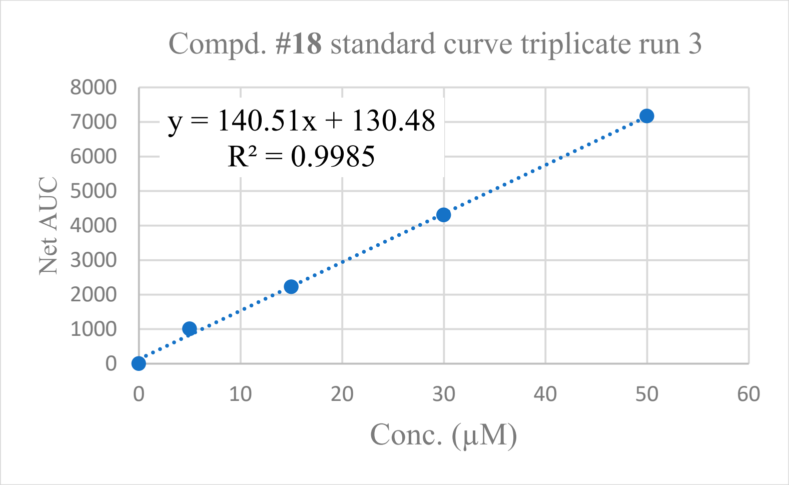
Compd. #18 standard curve triplicate run 3
y = 140.51x + 130.48
R² = 0.9985
Net AUC
Conc. (μM)
0
1000
2000
3000
4000
5000
6000
7000
8000
0
10
20
30
40
50
60

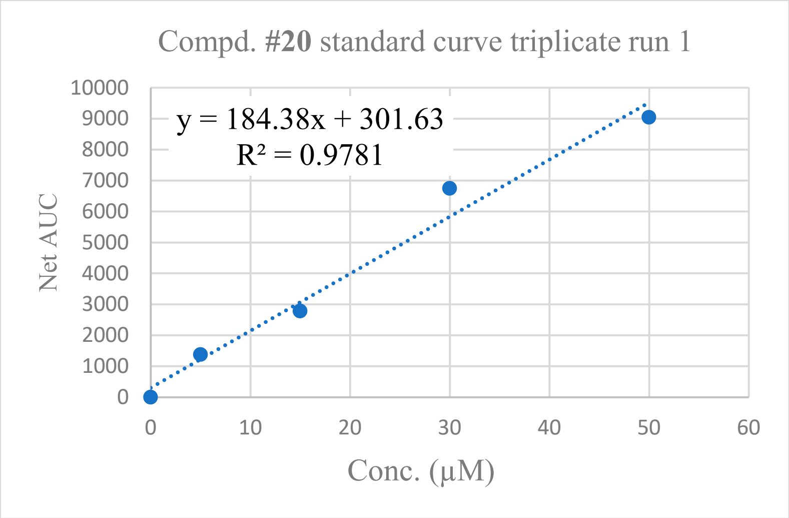
Compd. #20 standard curve triplicate run 1
y = 184.38x + 301.63
R² = 0.9781
Net AUC
Conc. (μM)
0
1000
2000
3000
4000
5000
6000
7000
8000
9000
10000
0
10
20
30
40
50
60

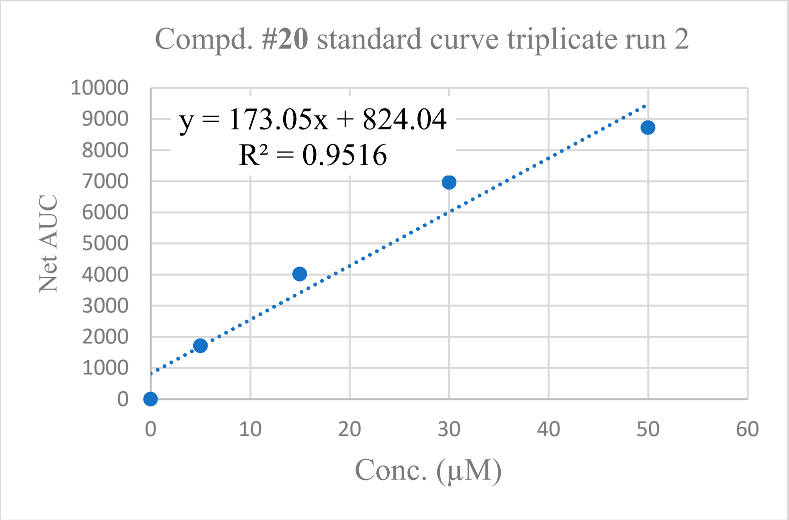
Compd. #20 standard curve triplicate run 2
y = 173.05x + 824.04
R² = 0.9516
Net AUC
Conc. (μM)
0
1000
2000
3000
4000
5000
6000
7000
8000
9000
10000
0
10
20
30
40
50
60

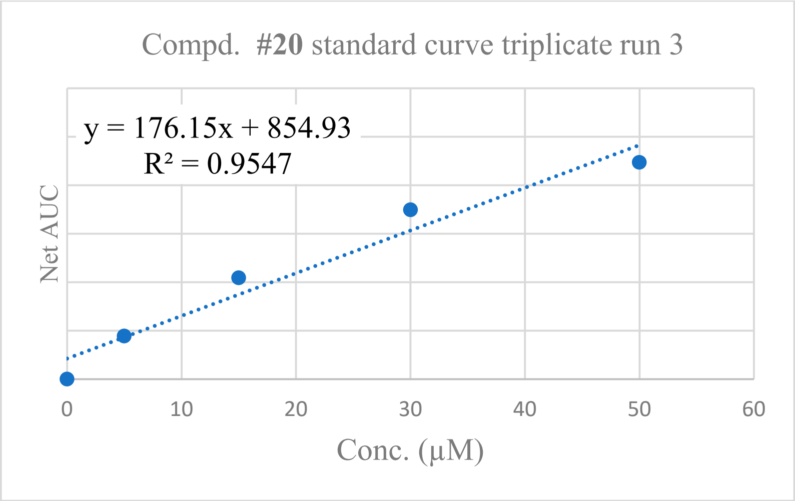
Compd. #20 standard curve triplicate run 3
y = 176.15x + 854.93
R² = 0.9547
Net AUC
0
10
20
30
40
50
60
Conc. (µM)
